# Single Crystals of Perylene Diimide‐Based Two‐Dimensional Covalent Organic Frameworks

**DOI:** 10.1002/adma.202523646

**Published:** 2026-05-23

**Authors:** Ling Zhang, Zixuan Chen, Lukas Mühlnickel, Lukas Sporrer, Jakub D. Jasinski, Megha Koottungal, Kevin Synnatschke, Paul‐Felix Jordan, Silvia Paasch, Eike Brunner, Zhehao Huang, Alexey Chernikov, Florian Auras

**Affiliations:** ^1^ Faculty of Chemistry and Food Chemistry TUD Dresden University of Technology Dresden Germany; ^2^ Max Planck Institute of Microstructure Physics Halle Germany; ^3^ School of Emergent Soft Matter Center for Electron Microscopy Guangdong Provincial Key Laboratory of Functional and Intelligent Hybrid Materials and Devices South China University of Technology Guangzhou China; ^4^ Institute of Applied Physics and Würzburg‐Dresden Cluster of Excellence ctd.qmat TUD Dresden University of Technology Dresden Germany; ^5^ State Key Laboratory of Luminescent Materials and Devices Guangdong Basic Research Center of Excellence for Energy and Information Polymer Materials Guangzhou China

**Keywords:** COFs, covalent organic frameworks, photoluminescence, single crystals

## Abstract

Two‐dimensional covalent organic frameworks (2D COFs) are crystalline porous polymers with highly tunable structural and electronic properties. Although single crystals of these materials are highly desirable for fundamental research and potential applications, their synthesis has so far been limited to only a few examples. We have developed a high‐temperature double‐modulator synthetic strategy that enables the growth of single crystals from fully dissolved precursors. We applied this approach to generate a series of perylene diimide (PDI)‐based 2D COFs. These materials crystallize as platelets with lateral dimensions reaching up to 50 µm for the biphenyl‐linked PDI(Me)_8_‐2P COF, providing a suitable platform for studying electronic processes via micro‐spectroscopy. Photoluminescence (PL) measurements revealed ultrafast monomer‐like emission together with a slower excimer‐related component. The availability of COF single crystals further enabled polarization‐dependent measurements, which revealed that the fast PL component is linearly polarized due to the parallel orientation of the PDI chromophores in the frameworks. These findings highlight the importance of COF single crystals for elucidating structure—photophysical property relationships of these intriguing materials.

## Graphical abstract

Synthesizing extended crystals of two‐dimensional covalent organic frameworks (2D COFs) has remained a major challenge. Here, we report a high‐temperature double‐modulator strategy that provides access to new perylene diimide (PDI)‐based COFs with crystal sizes of up to 50 µm. Owing to the parallel alignment of the PDI chromophores, the COF crystals exhibit linearly polarized photoluminescence.

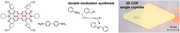

## 1 Introduction

COFs are crystalline and porous 2D or 3D polymers synthesized from (hetero‐)aromatic monomers via reversible condensation reactions [1, 2, 3, 4]. A wide range of functional building blocks enables the construction of COFs with tailored structural, electronic, and catalytic properties [5, 6, 7, 8, 9, 10, 11, 12], making these materials highly promising for applications in gas separation, energy storage, optoelectronics, catalysis, and sensing [13, 14, 15, 16, 17, 18, 19, 20, 21, 22, 23, 24].

These applications could strongly benefit from the exceptional structural precision of COFs, allowing for bespoke pore architectures, catalytic functionalities, or electronic characteristics. In reality, however, COF polymerizations under solvothermal conditions typically yield polycrystalline powders with domain sizes ranging from 1 µm to 100 nm, or even smaller [25, 26, 27]. The resulting high density of grain boundaries can severely impede pore accessibility, limit long‐range charge and energy transport, and induce electronic defects.

Recently, single crystals of imine‐linked 3D COFs have been achieved via modulator‐based synthetic strategies [28, 29]. In these approaches, monofunctional capping agents such as aniline are employed to suppress the rapid formation of amorphous precipitates [30]. The modulators then gradually exchange for the interconnecting monomers, enabling the controlled growth of high‐quality COF crystals from homogeneous solution [31].

The growth of 2D COF single crystals, however, is more challenging. Covalent in‐plane polymerization must be balanced with the non‐covalent assembly of COF layers, and both processes require effective error‐correction mechanisms under the selected synthesis conditions. Modulated syntheses using monofunctional amines have proven particularly effective at room temperature or slightly above [32, 33]. However, extended aromatic building blocks such as pyrenes and PDIs generally require elevated temperatures to achieve sufficient solubility and effective error correction during non‐covalent *π*‐stacking [34]. Recently, Dichtel and co‐workers developed a benzonitrile/benzoic acid/aniline reaction system that enabled the synthesis of high‐quality µm‐sized 2D COF crystals at 90°C–150°C [35, 36]. X‐ray scattering studies revealed that these crystals grow via a nonclassical crystallization process involving the fusion of smaller crystallites [37]. As a result, the frameworks exhibit excellent crystallinity within the *a*−*b* plane while still displaying various forms of stacking disorder, even within the same crystallite [38].

**FIGURE 1:**
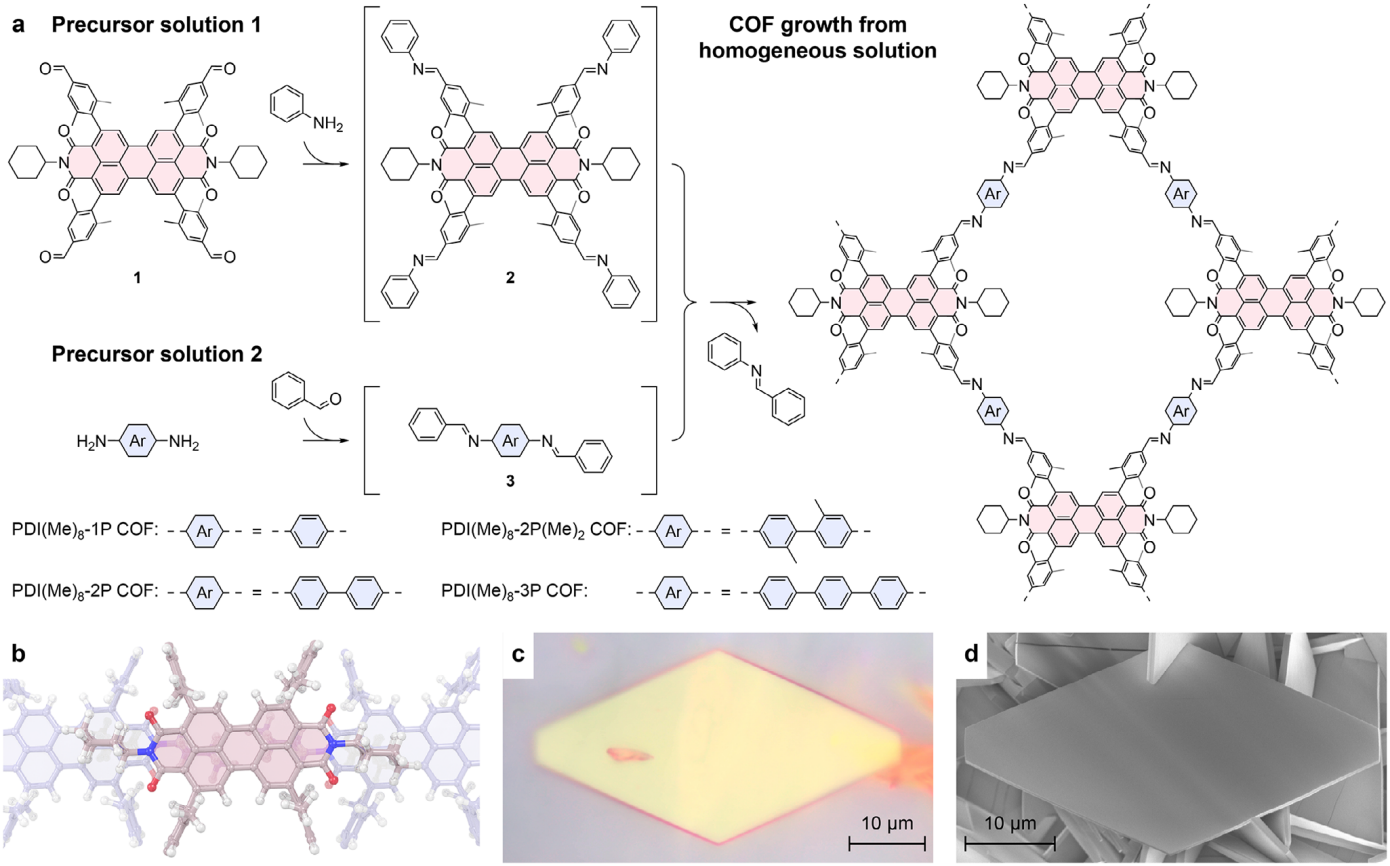
(a) Chemical structures and synthesis of the new 2D COF single crystals. Two separate precursor solutions are formed by reacting the aldehyde and amine building blocks with aniline and benzaldehyde, respectively. Injection of the diamine precursor solution at high temperature induces the COF growth from homogeneous solution, leading to well‐faceted COF platelets. (b) Illustration of the AB stacking of the PDI moieties. (c) Optical microscope image of a 48 × 26 µm PDI(Me)_8_‐2P COF crystal. (d) Scanning electron microscopy (SEM) image of a 46 × 25 × 0.8 µm PDI(Me)_8_‐2P COF crystal.

Here, we report the synthesis of imine‐linked 2D COF single crystals with sizes approaching 50 µm via an aniline/benzaldehyde double‐modulator approach. The COFs are constructed from a new PDI tetraaldehyde building block combined with linear aromatic diamines (Figure 1a). COF crystals are grown via a two‐step synthesis, where each monomer is first reacted separately with its corresponding modulator to form homogeneous precursor solutions. Injection of the modulator‐capped diamine into the PDI tetraaldehyde solution initiates COF polymerization, yielding rhombic platelets with lateral dimensions of up to 50 µm within 1–4 days (Figure 1c,d). Structure analysis via 3D electron diffraction (3DED) confirmed their single‐crystalline nature and revealed an uncommon AB layer sequence in which adjacent COF layers are displaced by half a unit cell along the crystallographic *a*‐axis. The COF single crystals contain rows of aligned PDI moieties, enabling the first investigation of orientation‐dependent optical properties in 2D COFs. We found that the photoluminescence of the frameworks is composed of a very fast monomer‐like emission that is linearly polarized along the *b*‐axis of the COF crystals, and a non‐polarized excimer‐related emission component.

## 2 Results and Discussion

### 2.1 Monomer Design

PDIs are among the most widely used organic dyes and have shown intriguing crystal structure‐dependent photophysical properties, such as controllable H‐ or J‐type aggregation, large Davydov splitting, and solid‐state lasing [39, 40, 41, 42, 43, 44]. Owing to their flat aromatic core and electrostatic interactions induced by the imide moieties, unsubstituted PDIs exhibit a strong tendency to form *π*‐stacked wires or needles [45, 46, 47]. As the synthesis of larger 2D COF single crystals requires a careful balance between the in‐plane and out‐of‐plane growth, we anticipated that strong *π*‐stacking interactions could dominate the crystallization process. We therefore designed a new PDI monomer bearing four 3,5‐dimethylbenzaldehyde substituents at the *ortho* positions (Figure 1a). The methyl groups are located above and below the PDI core [48], thereby preventing direct *π*–*π* contacts between adjacent PDIs while still providing sufficient space to support AB stacking motifs with an 11 Å displacement along the long molecular axis (Figure 1b).

### 2.2 Synthesis of COF Single Crystals

Inspired by recently reported COF syntheses using a benzonitrile/benzoic acid/aniline reaction system [35], and by the crystallization of 2D COFs using combinations of two modulators [49, 50, 51], we developed a high‐temperature double‐modulator approach (Figure 1a). Here, the PDI tetraaldehyde is reacted with excess aniline at 140°C to form a homogeneous solution (see the Experimental Section for detailed procedures). Meanwhile, the linear diamine monomer is protected with the second modulator, i.e., benzaldehyde, in a separate vial. The modulator‐capped diamine is then injected into the hot PDI solution to initiate COF growth from fully dissolved precursors via acid‐catalyzed imine exchange reactions. The reaction mixture is immediately transferred to a 120°C oven, where it turns cloudy after 2–4 h. The COF crystals continue to grow over 3–4 d until the final crystal dimensions are reached. The chemical structure of the polymers was confirmed by infrared (IR) and solid‐state nuclear magnetic resonance (ssNMR) spectroscopy (Figures S7 and S8). Control experiments using ^15^N‐labeled aniline confirmed that the modulator is fully replaced by the interconnecting diamine monomers (Figure S9).

During the optimization of our synthesis protocol for the PDI(Me)_8_‐1P COF, we found that the crystal size, shape, and quality depend strongly on the modulator‐to‐monomer ratio (Figure S1). Using only aniline yielded rounded crystals with an average diameter of approximately 1 µm and moderate crystallinity. Employing both aniline and benzaldehyde led to a significant improvement in crystal size and powder X‐ray diffraction (PXRD) intensities. Upon increasing the modulator content, the crystal morphology transitioned from rounded, intergrown crystallites to large, rhombic platelets. We found that the optimized conditions consisted of 6 equivalents each of aniline and benzaldehyde per aldehyde and amine functional group, respectively, yielding exceptionally crystalline, large, and well‐faceted platelets. Further increasing the amounts of modulators or deviating from the 1:1 aniline/benzaldehyde ratio led to smaller, very thin platelets with reduced crystallinity.

Additional key parameters affecting crystal growth are the solvent and reaction temperature (Figures S2 and S3). While benzonitrile produced large and thin platelets, chlorobenzene led to similarly crystalline but smaller, thicker, and more faceted crystals. In contrast, 1,2‐dichlorobenzene and nitrobenzene yielded very thin and heavily intergrown platelets of moderate crystallinity. The optimized temperature for the COF growth was 120°C. Both lower and higher temperatures produced smaller crystals with reduced crystallinity.

### 2.3 Single‐Crystal Structure of the PDI(Me)_8_‐1P COF

**FIGURE 2:**
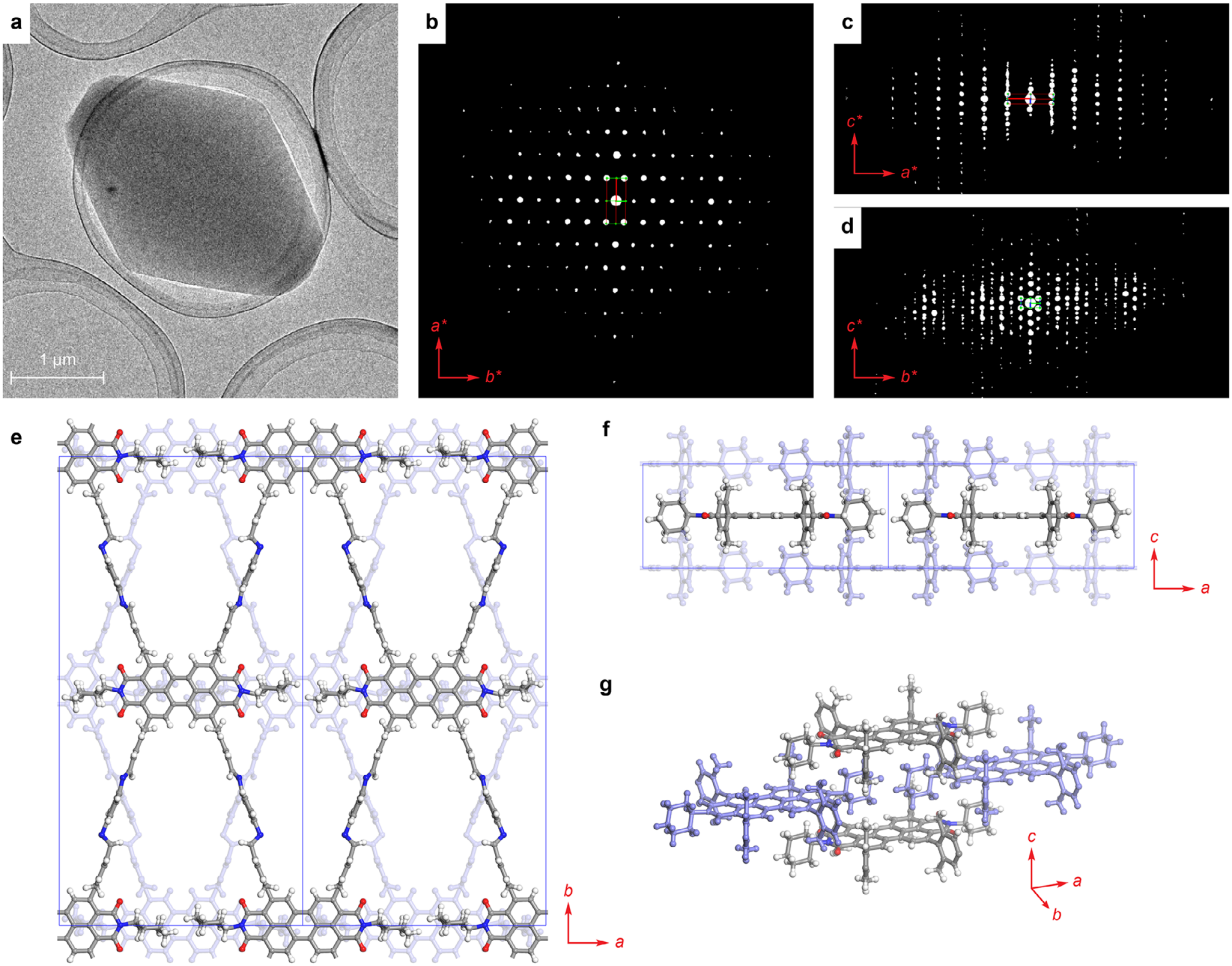
Single crystal electron diffraction analysis of the PDI(Me)_8_‐1P COF. (a) Transmission electron microscopy (TEM) image of the COF crystal used by 3DED. (b–d) 3D reciprocal lattice reconstructed from 3DED data, viewed along the *c* ^*^, *b* ^*^ and a^*^ directions, respectively. For clarity, the disorder of the cyclohexyl moieties and imine linkages is not shown. (e,f) COF structure viewed along the *c‐* and *b*‐axes, respectively. The COF consists of two layers that are shifted along *a* by 11.4 Å. (g) The PDIs assume a brick‐wall pattern in which the cyclohexyl moieties fit exactly into the grove defined by the PDI cores and methyl groups of the adjacent layers.

We employed 3DED, a technique developed for the structure analysis of nanocrystals [52], to determine the crystal structure of the PDI(Me)_8_‐1P COF. Crystals suitable for 3DED analysis were synthesized using 4 equivalents of both modulators and a reduced reaction time of 4 h to limit the crystal size to approximately 2 × 3 × 1 µm (Figure 2a). 3DED data were collected at 293 K to maintain consistency with the other measurements. The 3D reciprocal lattice indicates that the COF crystallizes in an orthorhombic system with the unit cell parameters of *a* = 22.848(5) Å, *b* = 44.066(9) Å, and *c* = 9.706(2) Å (Figure 2b–d and Figure S5). Its structure was solved and refined in the space group *Fmmm*.

As anticipated from the PDI monomer design, the COF possesses a double‐layer (AB) structure in which adjacent layers are displaced by 11.4 Å along the crystallographic *a*‐axis, corresponding to half a unit cell (Figure 2e,f). The cyclohexyl moieties are disordered with four possible orientations, and the imine‐linked bridges are disordered with eight possible orientations (for clarity, only one orientation is shown in Figure 2).

The eight methyl groups per PDI building block not only shield the PDI core from forming close *π*–*π* contacts, but also create a pocket that accommodates the cyclohexyl rings of the adjacent layers, leading to a geometrically interlocked AB arrangement of the PDIs (Figure 2g). We have shown previously that geometrically interlocking the COF layers can lead to highly crystalline and stable COFs [5, 25], and we ascribe the excellent crystallinity of the new PDI COFs, at least in part, to this well‐defined stacking.

The COF crystals exhibit a truncated rhombic prism morphology in which the crystallographic *b*‐axis is oriented along the longest crystal dimension, the *a*‐axis along the shorter dimension of the rhombic basal plane, and the *c*‐axis, i.e., the stacking direction, perpendicular to this plane. Accordingly, the crystallites are terminated by the (110), (010), and (001) facets.

### 2.4 Larger PDI COFs

Replacing 1,4‐phenylenediamine with longer linear diamines yielded the PDI(Me)_8_‐2P, PDI(Me)_8_‐2P(Me)_2_, and PDI(Me)_8_‐3P COFs. These frameworks display similarly high crystallinity and platelet‐like morphologies to the PDI(Me)_8_‐1P COF, but with larger unit cells (Figure S4). The crystal structures of these frameworks were determined via Rietveld refinement of the PXRD data.

**FIGURE 3:**
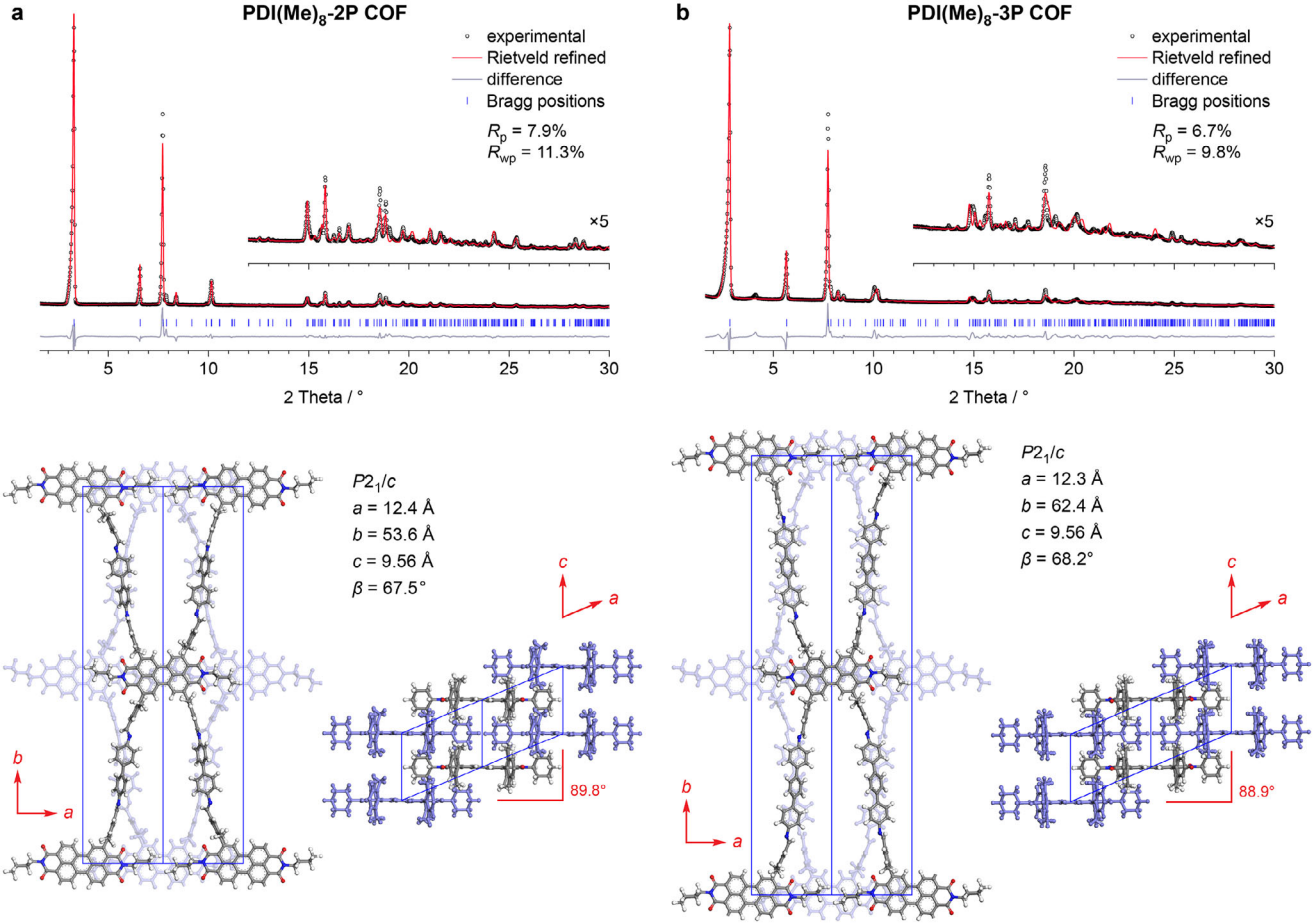
Structure of the larger PDI COFs. (a) PDI(Me)_8_‐2P COF. Top: Rietveld refinement of the PXRD data. Bottom: The COF has an AB stacking pattern with slit‐like micropores. (b) PDI(Me)_8_‐3P COF. Top: Rietveld refinement of the PXRD data. Bottom: The COF has an AB‐stacked structure with slit‐like micropores.

The PDI(Me)_8_‐2P COF forms particularly large platelets that can reach nearly 50 µm along their longest dimension, i.e., along the crystallographic *b*‐axis. (Figure 1c,d and Figure S4b), suggesting an optimal match between the steric requirements of both building blocks. Its crystal structure was solved in the monoclinic space group *P*2_1_/c (Figure 3a). The COF features the same double‐layer structure as the PDI(Me)_8_‐1P COF, but deviates slightly from orthorhombic symmetry, as the angle between the *c*‐axis and the PDI layers is 89.8° (Figure 3a, bottom).

The PDI(Me)_8_‐2P(Me)_2_ COF possesses a nearly identical unit cell and the same AB stacking pattern (Figure S6). Its crystallites, however, are smaller and less regularly shaped (Figure S4c). We attribute this to the additional steric demand imposed by the methyl groups, which likely introduces strain into the crystal lattice.

The even larger PDI(Me)_8_‐3P COF is isostructural with the latter two COFs but with a substantially elongated *b*‐axis due to the terphenyl linker (Figure 3b). The deviation from orthorhombic symmetry is again small, with an angle of 88.9° between the *c*‐axis and the PDI layers. The COF crystallizes as large, well‐faceted platelets with lateral dimensions reaching up to 33 µm along the crystallographic *b*‐axis (Figure S4d).

Due to the AB stacking, the COFs are expected to be microporous materials. This was confirmed by N_2_ sorption experiments, in which the COFs display type‐I isotherms and very narrow pore size distributions centered around 8 Å, corresponding to the wall‐to‐wall distances of the slit‐like pores (Figure S11).

### 2.5 Spectroscopic Analysis

Photophysical investigations of COFs have so far largely been limited to either powder samples or polycrystalline thin films. However, because 2D COFs are highly anisotropic, such measurements can provide only averaged characteristics. The new single‐crystal platelets now offer a unique opportunity to uncover their orientation‐resolved optical properties. We selected the PDI(Me)_8_‐2P COF as the primary candidate for these investigations, since this framework can be synthesized as particularly large and well‐faceted platelets with uniform thickness.

**FIGURE 4:**
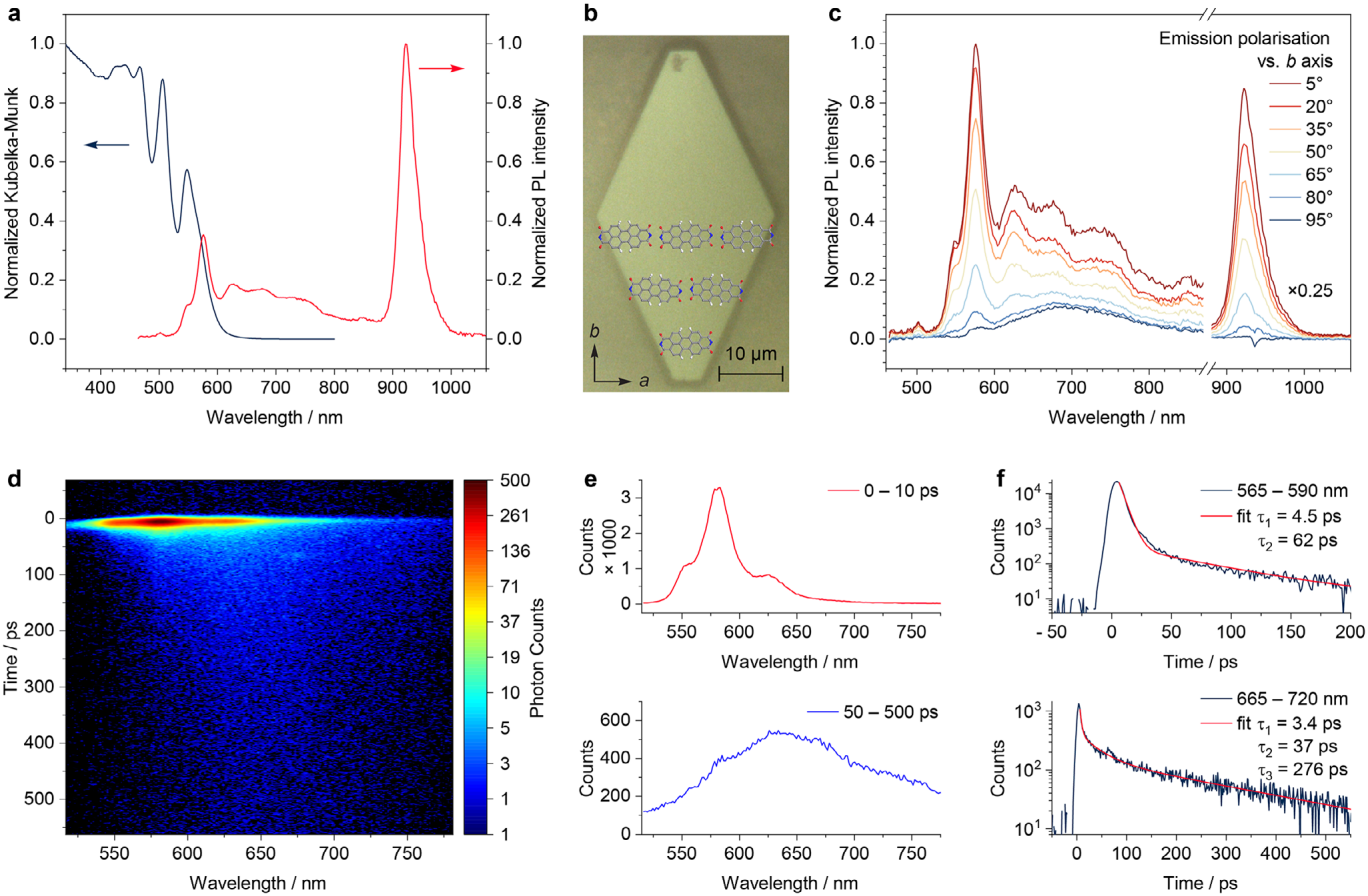
Spectroscopic investigation of PDI(Me)_8_‐2P COF single crystals. (a) Steady‐state diffuse reflectance spectrum of a COF powder sample (gray) and PL spectrum of a COF single crystal upon excitation at 420 nm. (b) Microscope image of a COF crystal used for the spectroscopy studies. The schematic illustrates the orientation of the PDI moieties. (c) Polarization‐dependent PL emission. Excitation at 420 nm was linearly polarized along the long dimension of the crystal, i.e., the crystallographic *b*‐axis. The emission polarizer was varied between 5° and 95° with respect to the COF *b*‐axis. (d) Time‐resolved PL of the COF, captured with a streak camera. (e) Decay‐associated spectra integrated from 0 to 10 ps (top) and 50–500 ps (bottom). (f) PL decay transients integrated from 565 to 590 nm (top) and 665–720 nm (bottom).

The absorption spectrum of this COF is dominated by the PDI moiety, which gives rise to an absorption band at 548 nm with well‐resolved vibronic sidebands at 507 and 467 nm (Figure 4a). The PL following excitation at 420 nm is dominated by a sharp emission band at 576 nm with vibronic side bands at 627 and 676 nm. This emission is reminiscent of the spectra of monomeric PDIs (Figure S12) and is rarely observed for densely aggregated chromophores [53]. The second contribution to the PL is a broad, featureless emission ranging from 550 to 850 nm. Similar PL is commonly observed for stacked H‐aggregated PDIs and has been attributed to excimer formation [54, 55]. A third PL feature is a sharp emission peak at 922 nm, which may arise from phosphorescence. *Ortho*‐substituted PDIs are known for fast and efficient intersystem crossing, and narrow phosphorescence emission at 950 nm has previously been observed for PDIs at low temperatures [46, 55].

We then studied the polarization‐dependent PL of a COF platelet. Excitation with linearly polarized light was most effective when the polarization direction was aligned with the long axis (*b*‐axis) of the COF crystals, i.e., perpendicular to the orientation of the PDI moieties (Figure 4b), but did not influence the shape of the spectrum. When polarization optics were also employed in the detection path, however, we found that different PL components could be distinguished (Figure 4c). While the broad excimer‐like PL is observed under all polarization angles, the monomer‐like PL and the sharp 922 nm feature are strongly polarized perpendicular to the PDI cores, i.e., the same direction that was most efficient for excitation.

Time‐resolved PL using a streak camera revealed fundamentally different decay dynamics for the different species (Figure 4d). The decay‐associated spectrum integrated from 0 to 10 ps after excitation is dominated by the monomer‐like PL (Figure 4e). This assignment is supported by the decay transients of the 565–590 nm range, which  feature a pronounced very fast component with τ_1_ = 4.5 ps (Figure 4f). The PL integrated from 50 to 500 ps consists primarily of broad emission from the excimer‐like species. This behavior is also reflected in the decay transients in the 665–720 nm range, which display a comparably long‐lived emission with τ_3_ = 276 ps (the faster component arises from the emission tail of the monomer‐like PL species).

These PL characteristics were highly uniform across the entire COF crystal. Spatially resolved PL maps revealed homogeneous PL intensities for both 576 and 922 nm emissions, with negligible variations in the emission wavelengths and energies (Figure S13).

Despite their near‐identical structures, the photophysical characteristics vary across the PDI COF series. The PDI(Me)_8_‐1P COF shows very similar PL spectra and emission time profiles to the PDI(Me)_8_‐2P COF (Figures S14 and S15). However, PL of the PDI(Me)_8_‐2P(Me)_2_ and PDI(Me)_8_‐3P COFs is more reminiscent of the PDI monomer with sharp singlet emission and very low contribution from excimer‐like states. Especially the time‐resolved PL of the PDI(Me)_8_‐3P COF shows hardly any spectral changes, indicating efficient suppression of the excimer formation (Figure S15c). Given the almost identical arrangement of the PDI moieties along *a* and *c* dimensions in all four COFs, we conclude that the observed differences arise from the coupling within the covalently linked COF layers. The shorter and more planar phenylenediamine and benzidine linkers may facilitate electronic interactions between adjacent PDIs and hence excimer formation, whereas the more twisted and longer tolidine and terphenyl diamines appear to act as efficient insulators, keeping the PDIs electronically separated.

### 2.6 Extension of the Modulator Strategy to Other COFs

In order to test the generality of our synthesis method, we applied the double‐modulator strategy to the widely studied TAPB‐DMPDA 2D COF (TAPB = 1,3,5‐tris(4‐aminophenyl)benzene, DMPDA = 2,5‐dimethoxyterephthalaldehyde). This material had previously been reported as thin platelets that grow via particle fusion [36, 37]. In contrast, our double‐modulator protocol yielded this COF as hexagonal prisms with widths of up to 3.3 µm and lengths of up to 6 µm (Figure S16). These results suggest that further optimization of the reaction conditions may enable the growth of even larger crystals.

## 3 Conclusion

We developed a new synthesis strategy for the growth of imine‐linked 2D COF single crystals with lateral dimensions of up to 50 µm. By employing two modulators to control the reactivity of both the amine and aldehyde building blocks, slow and controlled crystallization from initially homogeneous solution was achieved. We applied this strategy to synthesize a series of new PDI‐based COFs featuring a double‐layer (AB) structure. The parallel alignment of the PDI chromophores within the COF crystals suggested the presence of orientation‐dependent optical properties, which was confirmed by the observation of linearly polarized PL emission. These findings demonstrate the importance of COF single crystals and their unique advantages for elucidating the photophysics of these materials, and further highlight their potential for applications in optoelectronics, photocatalysis, and sensing.

## 4 Experimental Section

COF syntheses were performed in air using borosilicate glass culture tubes (12 mm × 100 mm, 7 mL volume) with polybutylene terephthalate (PBT) caps and polytetrafluoroethylene (PTFE)‐protected seals (DWK Life Sciences 261351155).

### 4.1 General Procedure for the COF Growth From Homogeneous Solution

- Solution 1. A culture tube was charged with PDI monomer 1 (8.6 mg, 8.0 µmol, 1.0 eq.), benzoic acid (122 mg, 1.0 mmol), benzonitrile (860 µL), and aniline (17.5 µL, 192 µmol, 6.0 eq. per aldehyde functional group). The reaction mixture was placed in a pre‐heated oil bath (140°C) and stirred until fully dissolved, followed by an additional 5 min of stirring.
- Solution 2. A second culture tube was charged with the respective linear diamine building block (16 µmol, 2.0 eq.), benzoic acid (31 mg, 0.25 mmol), benzonitrile (140 µL), and benzaldehyde (19.5 µL, 192 µmol, 6.0 eq. per amine functional group), and stirred at 140°C for 5 min.

Solution 2 was injected into Solution 1 at 140°C, and the resulting clear dark red solution was stirred at this temperature for 2 min. The stir bar was removed, and the culture tube was immediately placed in an oven at 120°C for 4 d. After cooling to room temperature, CHCl_3_ (2 mL) was added. The precipitate was collected by filtration, washed with CHCl_3_ (2 mL) and MeCN (2 mL), and dried under a stream of dry argon for 6 h.

Note: While 120°C was found to be the optimal temperature for COF growth (Figure S3), the higher temperature of 140°C was advantageous for preparing Solution 1 due to the very low solubility of the PDI monomer.

#### 4.1.1 PDI(Me)_8_‐1P COF

Synthesized following the above procedure using 1,4‐phenylenediamine (1.7 mg, 16 µmol, 2.0 eq.) as the linear building block. Average yield: 4.8 mg (49%). Optimization of the modulators was performed as described above, employing different amounts of aniline and benzaldehyde. Similarly, COF syntheses were performed using chlorobenzene, 1,2‐dichlorobenzene or nitrobenzene as the solvent. COF crystals suitable for structure analysis via 3DED were synthesized using 4.0 eq. of the modulators, and were removed from the oven after 4 h in order to obtain smaller high‐quality crystals with dimensions of about 2 × 3 × 1 µm along *a*, *b*, and *c*, respectively.

#### 4.1.2 PDI(Me)_8_‐2P COF

Synthesized following the above procedure using benzidine (2.9 mg, 16 µmol, 2.0 eq.) as the linear building block. Average yield: 4.9 mg (44%).

#### 4.1.3 Synthesis of the PDI(Me)_8_‐2P COF for ^15^N NMR

The COF was synthesized following the above procedure using ^15^N‐labelled aniline (17.5 µL, 192 µmol, 6.0 eq. per aldehyde functional group) as the modulator.

#### 4.1.4 PDI(Me)_8_‐2P(Me)_2_ COF

Synthesized following the above procedure using *m*‐tolidine (3.4 mg, 16 µmol, 2.0 eq.) as the linear building block. Average yield: 3.6 mg (31%).

#### 4.1.5 PDI(Me)_8_‐3P COF

Synthesized following the above procedure using 4,4″‐diamino‐*p*‐terphenyl (4.2 mg, 16 µmol, 2.0 eq.) as the linear building block. Average yield: 4.4 mg (36%).

### 4.2 Statistical Analysis

Data analysis was performed using OriginPro 2025.

#### 4.2.1 Pre‐Processing of Data

PXRD data for Rietveld refinement were averaged from 10 measurements of the same sample (30 min acquisition time per measurement). All other PXRD measurements are shown as unprocessed data.

PL data were binned (bin width 2 nm for steady‐state data and 4 pixels for streak camera data). To account for the spectral sensitivity of the spectrographs and ensure accurate relative PL intensities, the spectra acquired on the charge‐coupled device (CCD) detector were corrected using an intensity‐calibrated white‐light lamp. PL mapping was performed using custom‐made software for automated stage positioning and spectral acquisition. Relevant parameters, such as the signal intensity and energy position of the PL maxima, were extracted by fitting individual resonances with Gaussian peak functions.

#### 4.2.2 Sample Sizes

At least 5 identical batches of the COFs were prepared and analyzed to confirm reproducibility.

## Conflicts of Interest

The authors declare no conflicts of interest.

## Supporting information




**Supporting File 1**: adma73487‐sup‐0001‐SuppMat.pdf.

**Supporting File 2**: adma73487‐sup‐0002‐cif.zip.

## Data Availability

The data that support the findings of this study are available in the supplementary material of this article.
