# Supplementary material for: Single Crystals of Perylene Diimide‐Based Two‐Dimensional Covalent Organic Frameworks
Source: Adv Mater. 2026 May 23;38(36):e23646. doi: 10.1002/adma.202523646 (PMC13310095; doi:10.1002/adma.202523646)
Supplement: Supplementary file 1 — Supporting File 1: adma73487‐sup‐0001‐SuppMat.pdf. [file ADMA-38-e23646-s001.pdf]

SUPPLEMENTARY INFORMATION

Single Crystals of Perylene Diimide-Based Two-Dimensional Covalent Organic Frameworks

Ling Zhang,[1,2#] Zixuan Chen,[3#] Lukas Mühlnickel,[4] Lukas Sporrer,[1,2] Jakub D. Jasinski,[4] Megha Koottungal,[1] Kevin Synnatschke,[1] Paul-Felix Jordan,[1] Silvia Paasch,[1] Eike Brunner,[1] Zhehao Huang,[3,5] Alexey Chernikov,[4] Florian Auras[1,2]*

[1]Faculty of Chemistry and Food Chemistry, TUD Dresden University of Technology, 01217 Dresden, Germany

[2]Max Planck Institute of Microstructure Physics, 06120 Halle, Germany

[3]School of Emergent Soft Matter, Center for Electron Microscopy, Guangdong Provincial Key Laboratory of Functional and Intelligent Hybrid Materials and Devices, South China University of Technology, Guangzhou, China

[4]Institute of Applied Physics and Würzburg-Dresden Cluster of Excellence ctd.qmat, TUD Dresden University of Technology, 01062 Dresden, Germany

[5]State Key Laboratory of Luminescent Materials and Devices, Guangdong Basic Research Center of Excellence for Energy and Information Polymer Materials, Guangzhou, China

[#]These authors contributed equally *florian.auras@tu-dresden.de

Table of contents

A	Methods	2
B	Building block syntheses	3
C	COF syntheses	5
D	Impact of the modulators on morphology and crystallinity	6
E	Impact of the solvent on morphology and crystallinity	7
F	Impact of the reaction temperature	8
G	Morphology comparison of the different COFs	9
H	3D electron diffraction	10
I	Crystal structure of the PDI(Me)$_8$-2P(Me)$_2$ COF	11
J	IR spectroscopy	11
K	Solid-state NMR spectroscopy	12
L	Thermogravimetric analysis	13
M	N_2 sorption	14
N	Optical spectroscopy – PDI monomer	15
O	PL mapping of a PDI(Me)$_8$-2P COF crystal	15
P	PL spectroscopy – other PDI COFs	16
Q	TAPB-DMPDA COF	18
R	References	19

Abbreviations

Chemicals

dba	*trans,trans*-dibenzylideneacetone
cod	1,5-cyclooctadiene
DCB	1,2-dichlorobenzene
DCM	dichloromethane
DMSO	dimethyl sulfoxide
pin	pinacolato
PTFE	poly(tetrafluoroethylene)
SPhos	2-dicyclohexylphosphino-2′,6′-dimethoxybiphenyl

Other

BET	Brunauer-Emmett-Teller
eq.	equivalents
PSD	pore size distribution
PXRD	powder X-ray diffraction
TLC	thin layer chromatography

A. Methods

Nuclear magnetic resonance (NMR) spectra were recorded on Bruker Avance III 300 and Ascend 500 spectrometers. Chemical shifts are expressed in parts per million (δ scale) and are calibrated using the undeuterated solvent signals as an internal reference (^{1}H NMR: $CDCl_3$: 7.26, DMSO-d_6: 2.50; ^{13}C NMR: $CDCl_3$: 77.2, DMSO-d_6: 39.5).

Solid-state ^{13}C cross-polarisation magic angle spinning (CP-MAS) NMR spectra of the COFs were recorded with a Bruker Avance 300 MHz spectrometer at a resonance frequency of 75.5 MHz with a double resonance MAS NMR probe operating at MAS frequency of 12 kHz. ^{13}C C-MAS spectra of compound **2** were recorded with a Bruker Ascend 800 at a resonance frequency of 201.2 MHz and a MAS frequency of 16 kHz. Measurements were conducted via ramped ^{1}H-^{13}C cross-polarization (contact time: 4 ms, pulse repetition time: 3 s), with SPINAL ^{1}H-decoupling. ^{13}C chemical shifts were referenced with adamantane. The ACD/Labs software was used for prediction of ^{13}C NMR spectra.[1]

Solid-state ^{15}N MAS NMR spectra were recorded with Bruker Ascend 800 and Ascend 300 spectrometers at resonance frequencies of 81.1 MHz and 30.4 MHz, and MAS frequencies of 12 kHz and 15 kHz, respectively. Measurements were conducted via ramped ^{1}H-^{15}N cross-polarization (Ascend 800: contact time: 5 ms, pulse repetition time: 3 s; Ascend 300: contact time: 3.5 ms, pulse repetition time: 3 s). SPINAL ^{1}H-decoupling was applied. ^{15}N chemical shifts were referenced with glycine.

Fourier-transform infrared (FT-IR) spectra were measured with a Bruker Tensor II spectrometer equipped with a universal attenuated total reflection (ATR) accessory.

Powder X-ray diffraction (PXRD) patterns were measured in transmission geometry with a Stoe Stadi P diffractometer equipped with a primary monochromator for Cu $K_{\alpha 1}$ radiation (λ = 1.5406 Å) and a Dectris Mythen 1K Strip detector. The detector height was limited to 5 mm to reduce peak asymmetry. Powder samples were placed between two layers of Scotch tape (sample thickness ca. 0.3 mm, diameter 2 mm). Reflection positions and peak shape were calibrated against silver behenate.[2]

The **structure models of the COFs** were constructed using the Accelrys Materials Studio software package and were optimised via force field methods. Rietveld refinements were carried out as implemented in the Reflex module of Materials Studio. Pseudo-Voigt peak profiles were used, and peak asymmetry was corrected using the Finger-Cox-Jephcoat method.[3] Connolly surfaces were generated using an N_2-sized probe (r = 0.184 nm) at a 0.015 nm grid interval.[4]

Three-dimensional electron diffraction (3DED) was performed on a Talos F200X G2 FE-TEM (Thermo Fisher Scientific) operating at 200 kV with X-CFEG illumination. Data collection was carried out using a single-tilt tomography holder (±70°) and direct electron detector CheeTah T3 (512 × 512 pixels, pixel size 55 µm, Amsterdam Sci. Ins.), following the continuous rotation electron diffraction (cRED) protocol under low-dose conditions. The crystals were rotated continuously at a rate of 0.2906° s^{-1} during the data collection. The aperture used for cRED data collection was ~ 1.4 µm in diameter. The details of data collection are summarized in Tables S1. TEM images were acquired using a Ceta S CMOS camera (Thermo Fisher Scientific, 4096×4096 pixels, pixel size: 14 µm).

Structure solution and refinement. The cRED data had sufficient quality for ab intio structure determination of the COF. The framework structure was solved by using dual method implemented in SHELXT. The missing atoms were identified through electrostatic potential map in combination with model building. The structure was refined using SHELXL, and it converged to R_1 = 0.252. The details of data refinement are summarized in Tables S2.

Scanning electron microscopy (SEM) images were obtained with a Zeiss Gemini 500 microscope operated at 1.0 kV. COF samples were dispersed on carbon adhesive discs using a spatula (no solvents were used).

Nitrogen physisorption isotherms were recorded with a Micromeritics TriStar II Plus surface area and porosity analyzer. The samples were degassed at 333 K under high vacuum for 5 h prior to the measurements. The total pore volume was determined at p/p_0 = 0.9 in order to reduce the impact of textural porosity. Pore size distributions were calculated using Quantachrome Instruments software and the quenched solid density functional theory (QSDFT) equilibrium (desorption) model for N_2 on carbon with slit/cylindrical pores. Applying the adsorption model instead resulted in almost identical pore size distributions, confirming the robustness of the data analysis.

UV-Vis-NIR absorption and **diffuse reflectance** spectra were recorded on an Agilent Cary 5000 spectrometer equipped with a 150 mm InGaAs integrating sphere. COF samples were placed at the reflectance port inside the integrating sphere and held in position with adhesive tape. Reflectance data were converted using the Kubelka-Munk equation.

Steady-state and time-resolved room-temperature photoluminescence (PL) spectra of COF single crystals were recorded using a pulsed laser source with an excitation wavelength of 420 nm (photon energy 2.95 eV). The excitation light was generated via second harmonic generation pumped by an 80 MHz titanium sapphire laser with a pulse length of 140 fs and a tuned wavelength of 840 nm. The excitation laser was directed into an inverted microscope and focused onto the sample through a 60x glass-corrected objective (NA = 0.7), resulting in a spot with a full-width-at-half-maximum of approximately 0.7 µm. For polarization-resolved excitation measurements a linear polarizer in combination with a half-wave and quarter-wave plate were inserted into the beam path before the microscope. The quarter-wave plate was used to compensate for polarization changes caused by the beam splitter inside the microscope. The excitation fluence was set to 1 µJ cm^{-2} for polarization-resolved measurements, 3 µJ cm^{-2} for time-resolved and to 7 µJ cm^{-2} for mapping on the same sample spot. The COF sample was mounted inside a micro cryostat on an *x-y* translation stage and kept under high vacuum conditions to avoid oxygen-induced degradation. In the detection path, the emitted PL was guided through spectral filters to remove the excitation laser. For polarization-resolved measurements, the same polarization optics as described above for the excitation path were placed in reverse order. The PL signal was focused onto the entrance slit of a spectrograph, dispersed using a 50 l mm^{-1} grating, and detected either by a charge-coupled device (CCD) camera for spectrally resolved measurements or by a streak camera for time-resolved measurements.

B. Building block syntheses

All syntheses were performed in oven-dried glassware under argon atmosphere using standard Schlenk and glovebox techniques. Reagents and solvents were obtained in high-purity grades from commercial suppliers and were degassed and saturated with argon prior to use. Flash column chromatography was performed using silica gel (Acros Organics, 60 Å, 40 – 60 µm) and was continuously monitored via thin layer chromatography (TLC) using silica gel coated aluminium plates (Merck, 60 Å, F254). Benzidine was purified by column chromatography (silica gel, cyclohexane/EtOAc 1:1).

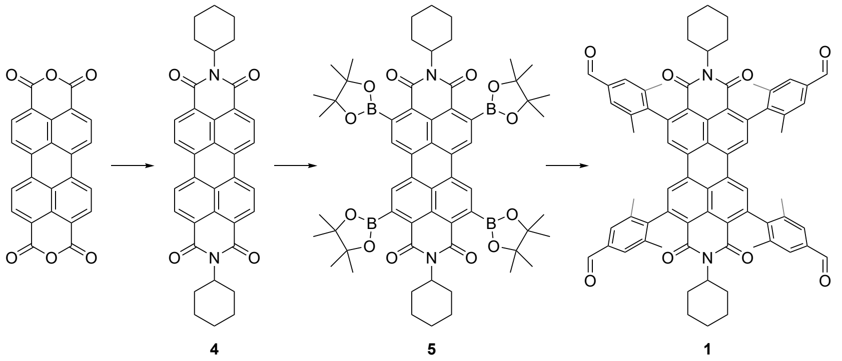

4 5 1

Scheme S1. Synthesis of the PDI building block.

***N,N′*-Dicyclohexyl-3,4,9,10-perylenedicarboximide (4)**

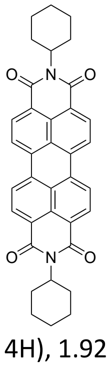

A mixture of perylene-3,4,9,10-tetracarboxylic dianhydride (1.57 g, 4.00 mmol, 1.0 eq.), $Zn(OAc)_2 \times 2\ H_2O$ (878 mg, 4.00 mmol, 1.0 eq.), cyclohexylamine (3.65 mL, 32.0 mmol, 8.0 eq.) and imidazole (32 g) was stirred at 130 °C for 16 h. The resulting suspension was diluted with 40 mL of chloroform while still hot, transferred to a 600 mL beaker, adjusted to pH 1 by addition of 2M aqueous HCl (ca. 300 mL), and stirred for 30 min. The precipitate was collected by filtration, washed with H_2O, and re-dispersed in a mixture of acetone and H_2O (95:5). After stirring for 4 h, the solids were collected by filtration, washed successively with H_2O, acetone, and Et_2O, and dried under high vacuum to yield the title compound as a dark red powder (2180 mg, 3.93 mmol, 98%).

^{1}H NMR (300 MHz, $CDCl_3$): δ 8.67 (d, J = 8.0 Hz, 4H), 8.61 (d, J = 8.1 Hz, 4H), 5.12 – 4.98 (m, 2H), 2.65 – 2.50 (m, 4H), 1.92 (d, J = 12.5 Hz, 4H), 1.84 – 1.71 (m, 6H), 1.52 – 1.25 (m, 6H).

Compound 5

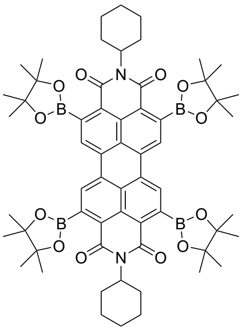

Adapted from literature.[5] A reaction mixture containing PDI **4** (1.64 g, 3.00 mmol, 1.0 eq.), $[Ir(OMe)(cod)]_2$ (99 mg, 0.15 mmol, 5%), $P(C_6F_5)_3$ (319 mg, 0.600 mmol, 20%) and $(Bpin)_2$ (6.09 g, 24.0 mmol, 8.0 eq.) in 1,4-dioxane (60 mL) and 1,3,5-trichlorobenzene (60 g) was stirred at 110 °C for 2 d. After cooling to room temperature, all volatiles were removed under reduced pressure (trichlorobenzene was only partially removed in this step). The crude product was purified by column chromatography (silica gel, gradient DCM + 5% EtOAc to DCM + 10% EtOAc). All product-containing fractions were collected and dried initially under reduced pressure, then high vacuum at 60 °C for 2 h. The residue was dissolved in DCM (50 mL) and cyclohexane (100 mL), and concentrated under reduced pressure to about 30 mL. The resulting precipitate was collected by filtration, washed with cyclohexane (15 mL), and dried under high vacuum to afford the title compound as a bright red powder (1659 mg, 1.57 mmol, 52%).

^{1}H NMR (300 MHz, $CDCl_3$): δ 8.51 (s, 4H), 5.01 – 4.87 (m, 2H), 2.60 – 2.43 (m, 4H), 1.92 – 1.68 (m, 10H), 1.56 (s, 48H), 1.51 – 1.18 (m, 6H).

^{13}C NMR (76 MHz, $CDCl_3$): δ 165.7, 133.3, 128.0, 127.2, 126.8, 126.0, 84.7, 54.3, 29.3, 27.1, 26.6, 25.2. The C-B signal is not observed due to quadrupolar broadening.

PDI monomer 1

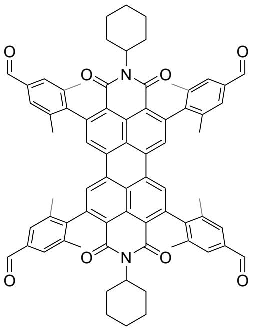

A solution containing PDI **5** (884 mg, 0.835 mmol, 1.0 eq.), 4-bromo-3,5-dimethylbenzaldehyde (1.07 g, 5.01 mmol, 6.0 eq.), K_2CO_3 (923 mg, 6.68 mmol, 8.0 eq.), $Pd_2(dba)_3$ (76 mg, 0.084 mmol, 10%) and SPhos (69 mg, 0.167 mmol, 20%) in a mixture of *o*-xylene (12.8 mL) and H_2O (3.2 mL) was stirred at 100 °C for 2 d. After cooling to room temperature, the crude product was mixed with H_2O and a few drops of brine, and extracted with $CHCl_3$. The combined organic phases were dried over $MgSO_4$ and concentrated under reduced pressure. The product was purified by column chromatography (silica gel, gradient $CHCl_3$ + 5% EtOAc to $CHCl_3$ + 10% EtOAc). The combined product fractions were concentrated under reduced pressure to about 50 mL to induce precipitation of the product. The solids were collected by filtration, washed with Et_2O and dried under high vacuum to yield the title compound as a bright red powder (442 mg, 0.409 mmol, 49%).

^{1}H NMR (500 MHz, $CDCl_3$): δ 10.04 (s, 4H), 8.12 (s, 4H), 7.74 (s, 8H), 4.71 (t, J = 12.2 Hz, 2H), 2.25 (q, J = 13.5 Hz, 4H), 2.12 (s, 24H), 1.71 (d, J = 12.9 Hz, 4H), 1.54 (d, J = 12.9 Hz, 4H), 1.32 – 1.20 (m, 6H), 1.15 (t, J = 12.9 Hz, 2H).

^{13}C NMR (126 MHz, $CDCl_3$): δ 192.2, 162.4, 148.3, 145.9, 135.5, 134.9, 133.8, 130.9, 129.2, 125.8, 125.7, 121.8, 54.0, 28.6, 26.2, 24.8, 20.8.

Compound 2 (^{15}N labelled)

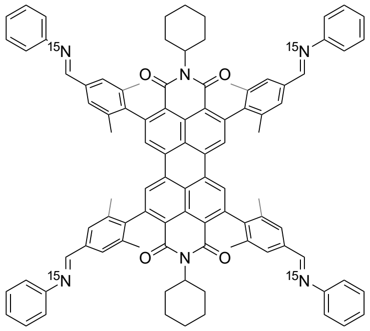

A culture tube was charged with PDI monomer **1** (8.6 mg, 8.0 µmol, 1.0 eq.), benzoic acid (122 mg, 1.0 mmol), benzonitrile (860 µL) and ^{15}N labelled aniline (98 atom % ^{15}N, Merck) (17.5 µL, 192 µmol, 6.0 eq. per aldehyde functional group). The reaction mixture was placed in a pre-heated oil bath (140 °C) and stirred for 30 min. After cooling to room temperature, the solution was mixed with 5 mL of MeCN, resulting in precipitation of the product. The solids were collected by filtration, washed with MeCN (10 mL) and $CHCl_3$ (10 mL), and dried under high vacuum to yield the title compound as a dark red solid. See Figure S9 for spectroscopic characterisation.

C. Additional COF syntheses

COF syntheses were performed in air using borosilicate glass culture tubes (12 mm × 100 mm, 7 mL volume) with PBT caps and PTFE-protected seal (DWK Life Sciences 261351155).

TAPB-DMPDA COF.[6]

Solution 1. A culture tube was charged with 1,3,5-tris(4-aminophenyl)benzene (10.5 mg, 30 µmol, 1.0 eq.), benzoic acid (92 mg, 0.75 mmol), benzonitrile (600 µL) and benzaldehyde (11.4 µL, 113 µmol, 1.25 eq. per amine functional group). The reaction mixture was placed in a pre-heated oil bath (120 °C) and stirred for 2 min.

Solution 2. A second culture tube was charged with 2,5-dimethoxyterephthalaldehyde (8.7 mg, 45 µmol, 1.5 eq.), benzoic acid (61 mg, 0.5 mmol), benzonitrile (400 µL), and aniline (10.3 µL, 113 µmol, 1.25 eq. per aldehyde functional group), and stirred at 120 °C for 2 min.

Solution 2 was injected into solution 1 at 120 °C, and the resulting clear yellow solution was stirred for 2 min. The stir bar was removed, and the culture tube was immediately placed in an oven at 120 °C for 20 h. After cooling to room temperature, 2 mL of MeCN was added. The precipitate was collected by filtration, washed with MeCN (2 mL), and dried in air for 6 h.

D. Impact of the modulators on morphology and crystallinity

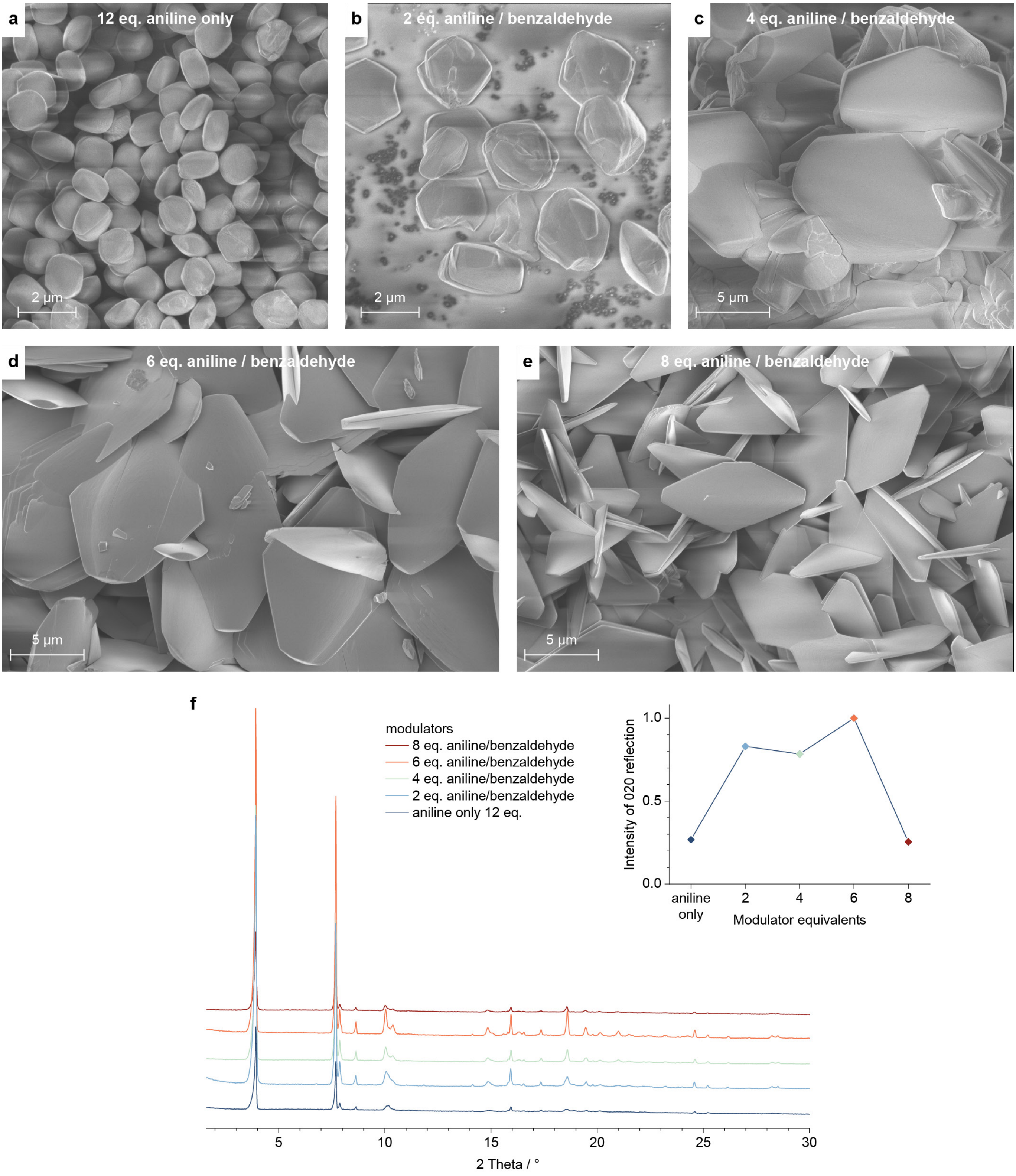

Figure S1. SEM micrographs of the PDI(Me)$_8$-1P COF synthesized using different amounts of the modulator(s). (**a**) Using only aniline leads to small, rounded crystals of medium crystallinity. Smaller amounts of aniline led to rapid formation of amorphous precipitates, whereas even larger excess of aniline did not yield any solid products. (**b**-**e**) With increasing excess of modulators per amine/aldehyde functional group, the COF crystals become larger and their shape transitions from rounded to faceted microplatelets. (**f**) Corresponding PXRD patterns of the COF samples. The highest crystal quality is obtained with 6 eq. of both modulators, whereas using larger excess leads to reduced crystallinity despite the very regular and faceted morphology. The inset shows the relative intensities of the 020 reflection at 2θ = 3.92°.

E. Impact of the solvent on morphology and crystallinity

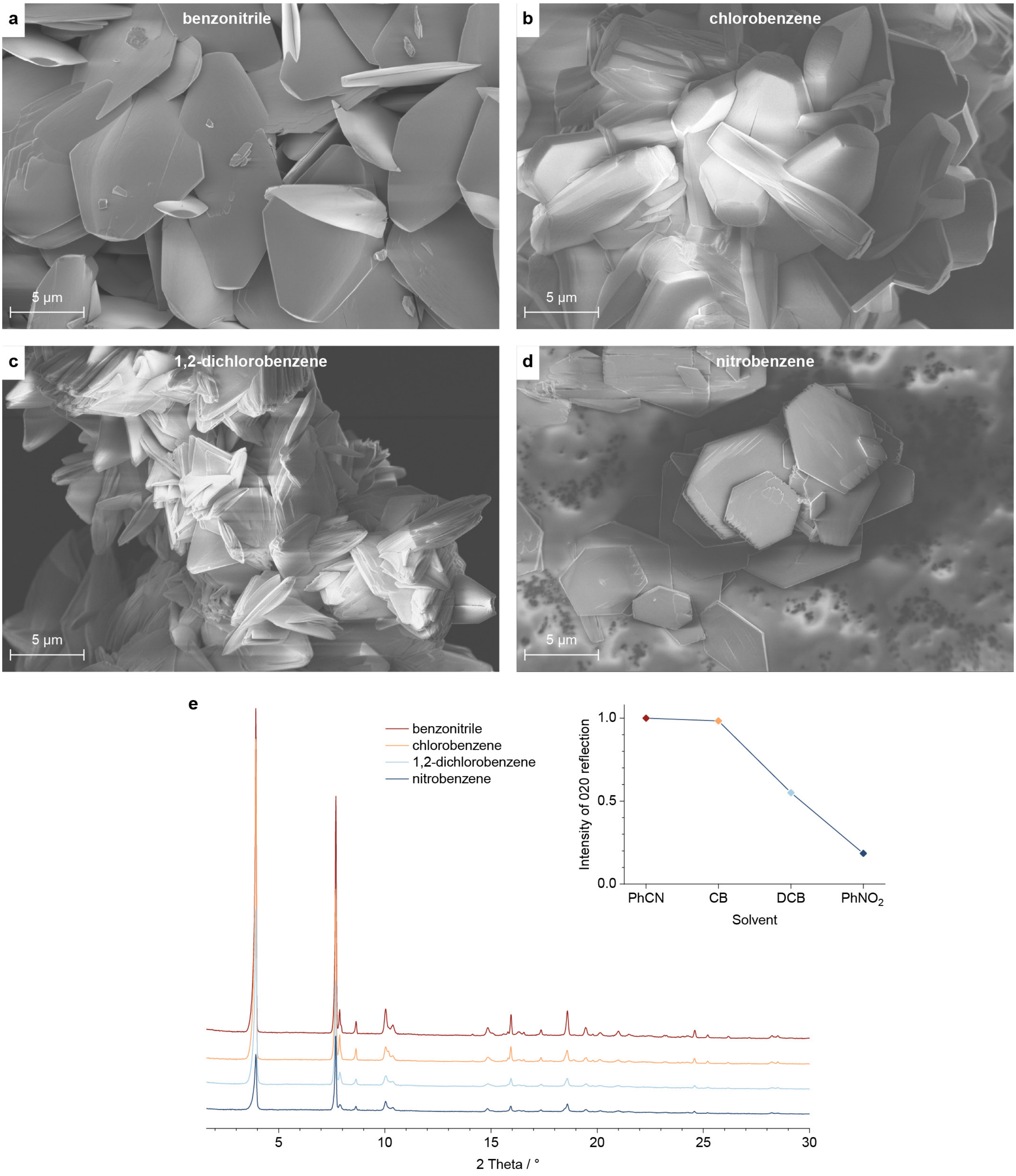

Figure S2. SEM micrographs of the PDI(Me)$_8$-1P COF synthesized in different solvents. (**a**) benzonitrile (same image as Figure S1d, added here for completeness), (**b**) chlorobenzene, (**c**) 1,2-dichlorobenzene, (**d**) nitrobenzene. (**e**) PXRD patterns of the COFs. The frameworks synthesized in benzonitrile and chlorobenzene have similar peak intensities, but the higher-angle reflections of the benzonitrile sample are sharper, indicating higher crystallinity. The inset shows the relative intensities of the 020 reflection at 2θ = 3.92°.

We noted that the reaction mixture remains visually clear for about 4 h when PhCN is used, whereas the other solvents lead to significantly faster precipitation. This could be due to slower imine exchange in PhCN (the nitrile reduces the protonation strength of the PhCOOH catalyst), or increased colloidal stability of the growing COF particles, or both. As both factors are key for growing high quality crystals, this could explain why PhCN performs best for all COFs in this study.

F. Impact of the reaction temperature

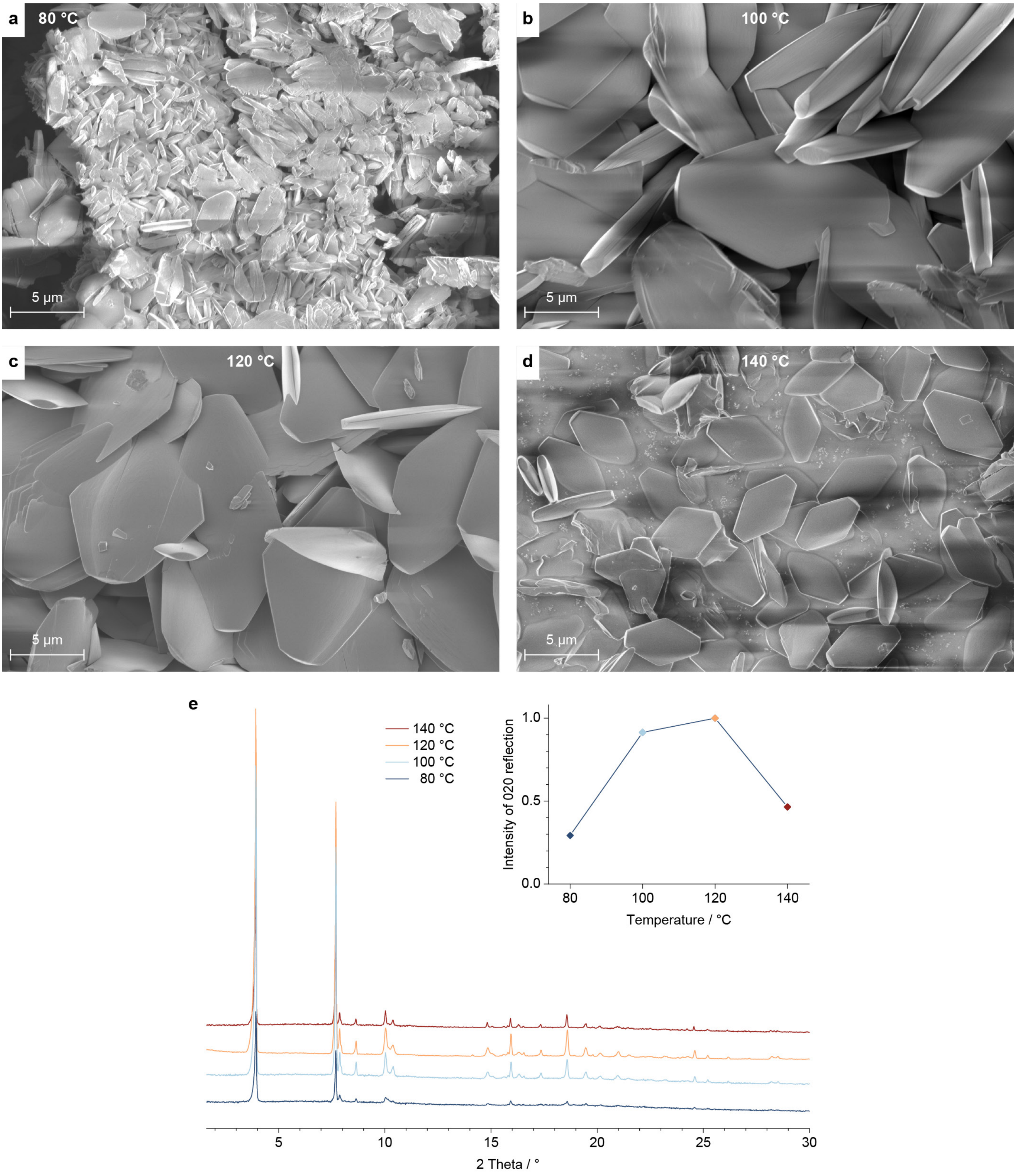

Figure S3. SEM micrographs of the PDI(Me)$_8$-1P COF synthesized at different temperatures. (**a**) 80 °C, (**b**) 100 °C, (**c**) 120 °C (same image as Figure S1d, added here for completeness), (**d**) 140 °C. (**e**) Corresponding PXRD patterns of the COFs, confirming that the highest crystallinity is achieved at 120 °C. The inset shows the relative intensities of the 020 reflection at 2θ = 3.92°.

G. Morphology comparison of the different COFs

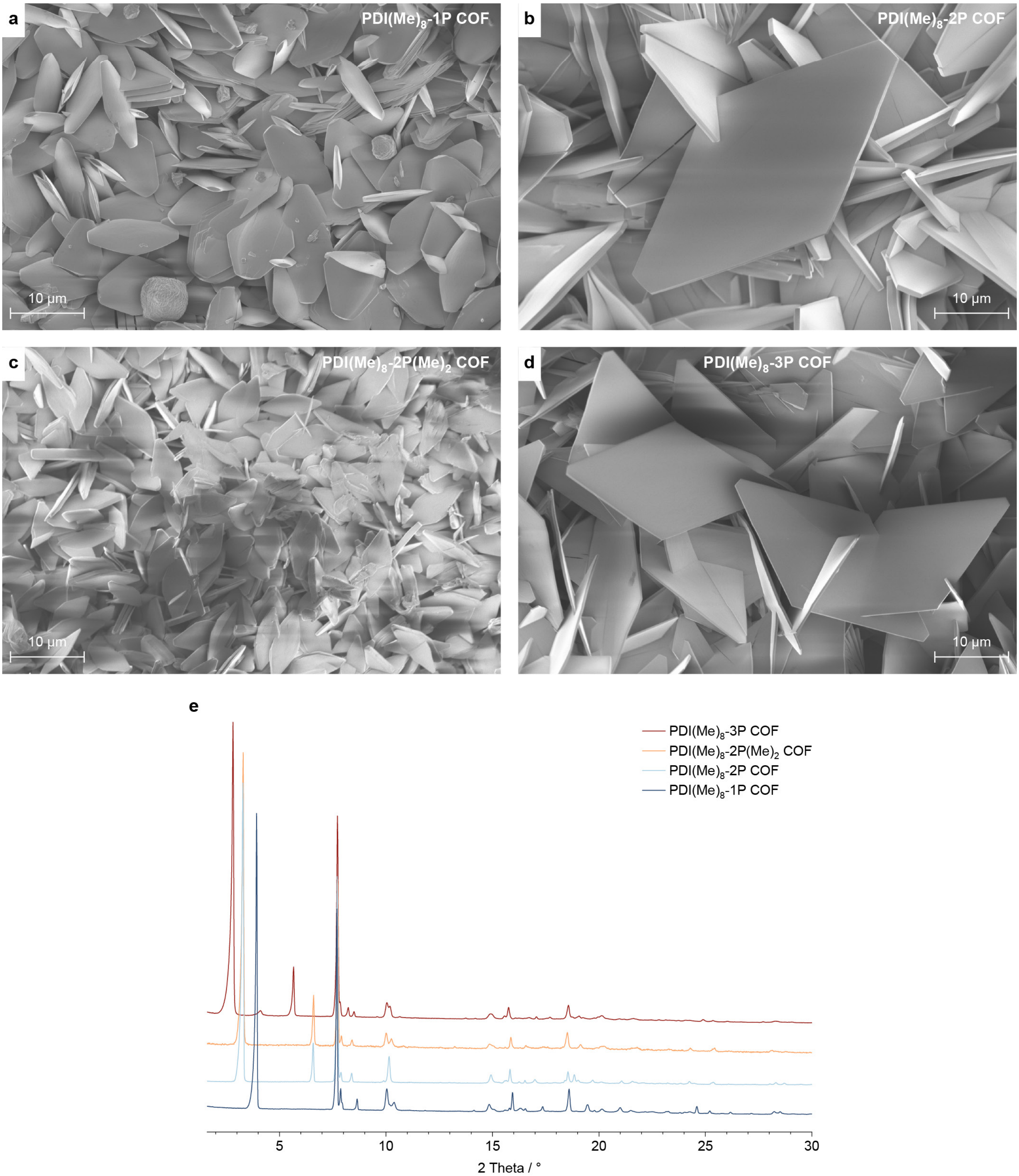

Figure S4. Comparison of the PDI COFs with different linear building blocks. (**a**) The PDI$(Me)_8$-1P COF forms 10 – 15 µm long platelets. (**b**) The PDI$(Me)_8$-2P COF crystallizes as very large platelets of up to 46 × 25 × 0.8 µm. The long axis of the platelets corresponds to the crystallographic *b* axis. (**c**) Despite its almost identical structure, the PDI$(Me)_8$-2P$(Me)_2$ COF forms smaller platelets with less regular shape. (**d**) The PDI$(Me)_8$-3P COF crystallizes as large and thin platelets of up to 33 × 17 × 0.4 µm. (**e**) Comparison of the PXRD patterns of the four COFs.

H. 3D electron diffraction

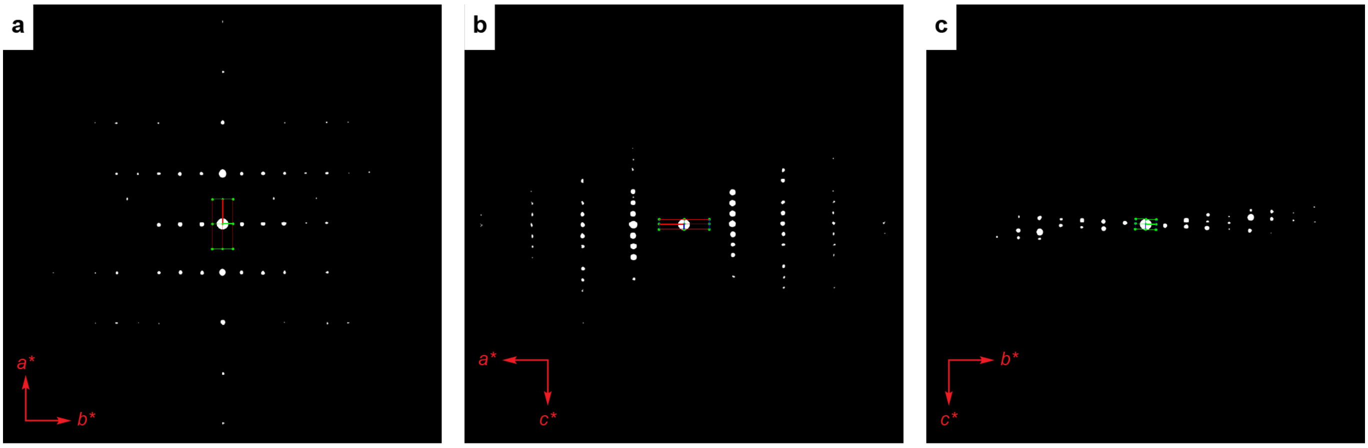

Figure S5. 2D slice cuts from the reconstructed 3D reciprocal lattice showing the (**a**) hk0, (**b**) h0l, and (**c**) 0kl planes of PDI(Me)$_8$-1P COF.

Table S1. Single crystal data collection parameters for the PDI(Me)$_8$-1P COF.

Temperature (K)	293(2)
Wavelength (Å)	Electron (λ = 0.02508 Å)
Tilt range (°)	55.99 to -48.35
Tilt rate (° s^{-1})	0.2906
Exposure time per frame (s)	0.998
Total number of frames	360
Data collection time (s)	359.05
Beam current (pA)	< 0.01

Table S2. Crystallographic details of the PDI(Me)$_8$-1P COF.

Name	PDI(Me)$_8$-1P COF
Chemical formula	$C_{42}H_{35}N_3O_2$
Formula weight	613.76
Crystal system	Orthorhombic
Space group	*Fmmm*
a (Å)	22.848(5)
b (Å)	44.066(9)
c (Å)	9.706(2)
Z	8
Cell volume (Å^3)	9772(3)
Completeness	0.765
Resolution (Å)	1.15
No. of reflections (all unique)	3680
No. of reflections (I>2σ(I))	741
R_{int}	0.252
R_1 (I>2σ(I))	0.252
R_1 (all reflections)	0.369
Goof	2.032

I. Crystal structure of the PDI(Me)$_8$-2P(Me)$_2$ COF

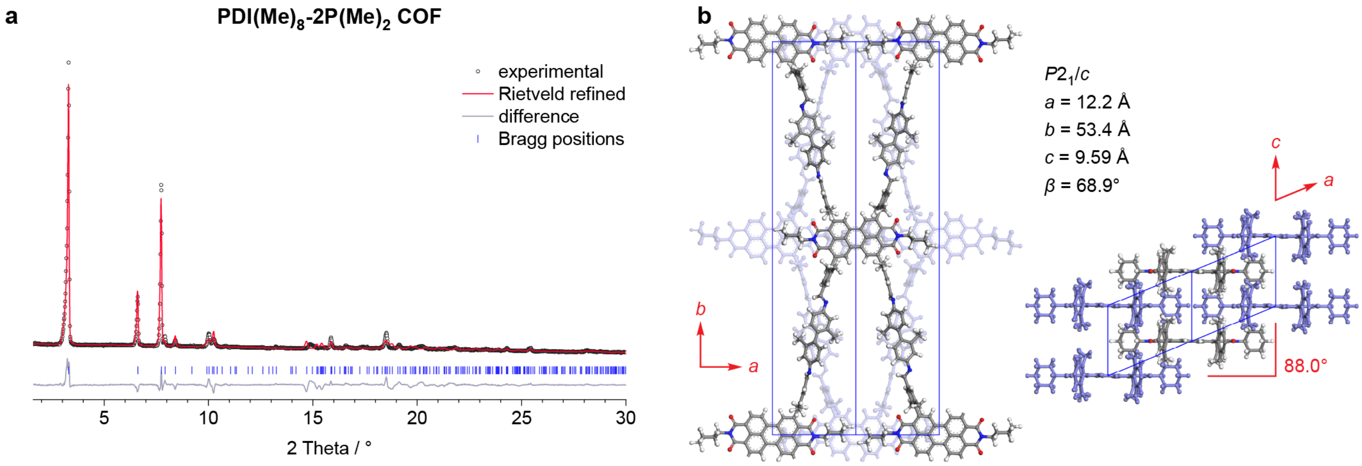

Figure S6. Crystal structure of the PDI(Me)$_8$-2P(Me)$_2$ COF. (**a**) Rietveld refinement of the PXRD data. R_p = 8.0%, R_{wp} = 12.3%. (**b**) The COF has an AB stacking pattern with slit-like micropores.

J. IR spectroscopy

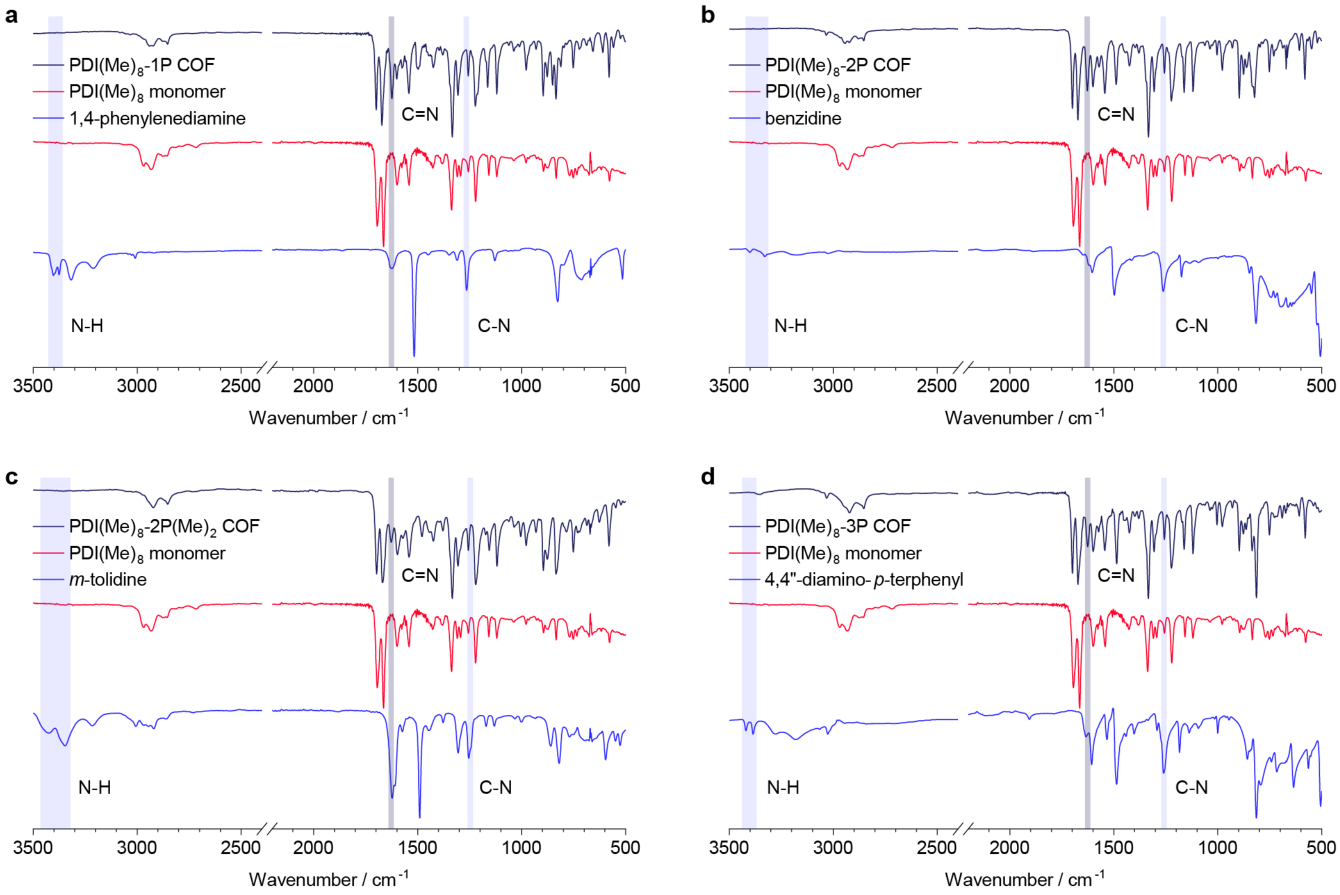

Figure S7. IR spectra of the PDI COFs (black) and the corresponding starting materials (red and blue). The characteristic signals of the amine and imine functional groups are highlighted. Successful formation of the imine-linked COFs is confirmed by the absence of the -NH_2 signals, and the appearance of the C=N signal at 1626 cm^{-1}.

K. Solid-state NMR spectroscopy

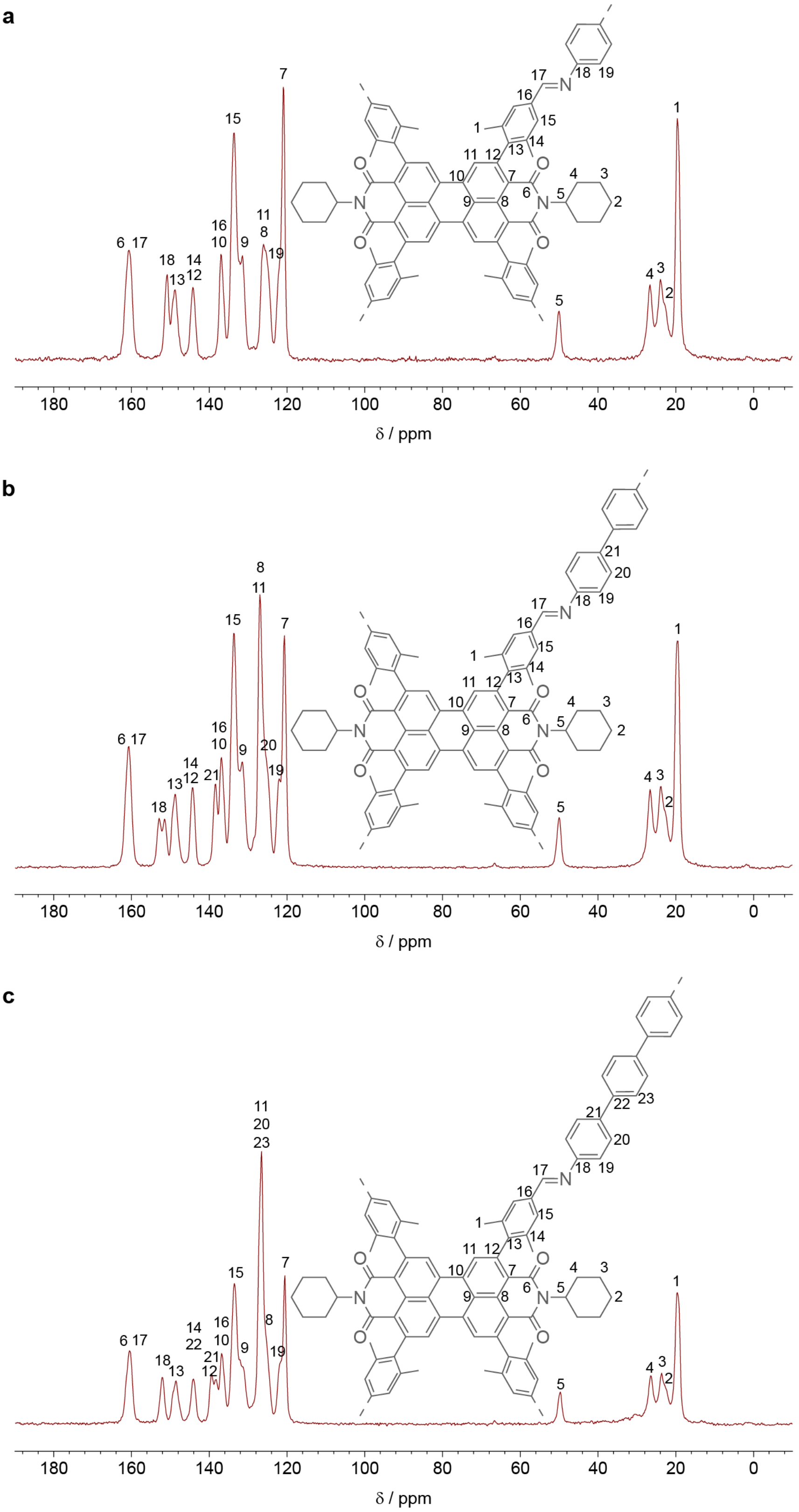

Figure S8. ^{13}C CP-MAS NMR spectra of (**a**) PDI(Me)$_8$-1P COF, (**b**) PDI(Me)$_8$-2P COF and (**c**) PDI(Me)$_8$-3P COF. The sharp and well-resolved signals highlight the exceptional crystallinity of the PDI COFs with exactly defined chemical environments of all moieties.

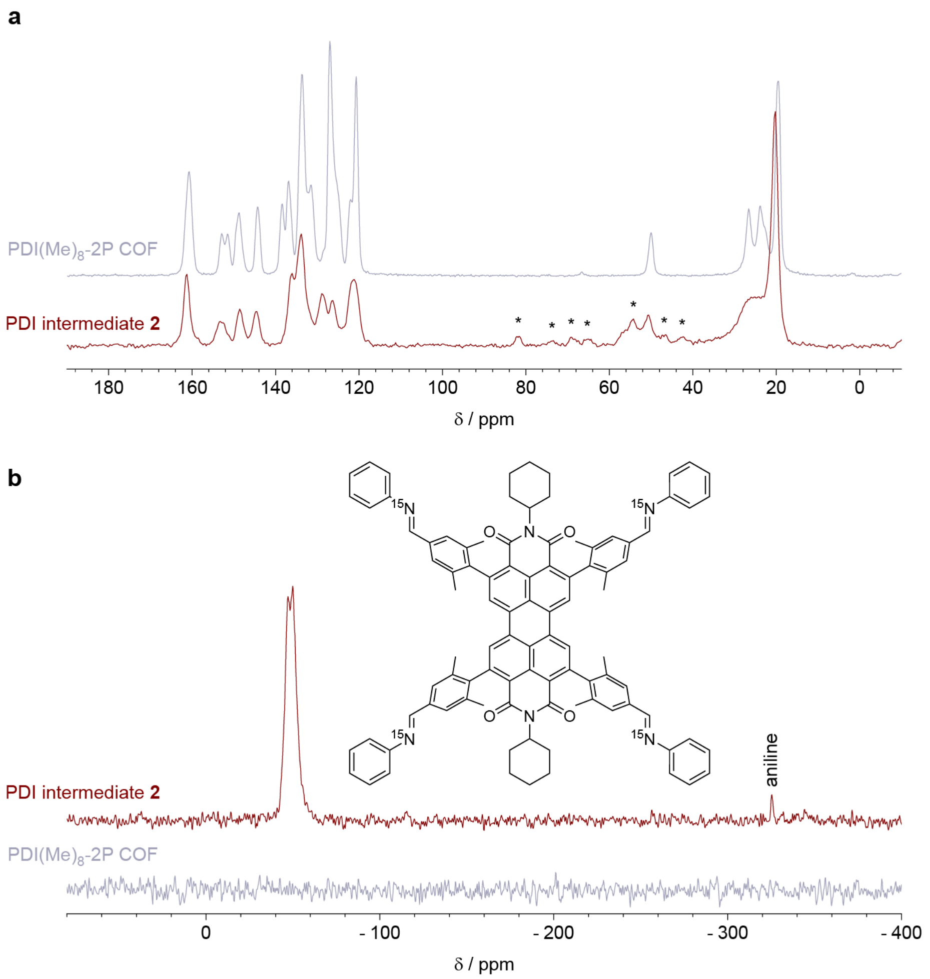

Figure S9. (**a**) Comparison of the ^{13}C CP-MAS NMR spectra of the aniline-capped intermediate **2** and the PDI(Me)$_8$-2P COF. Rotational side bands are marked with asterisks. (**b**) ^{15}N CP-MAS NMR spectra of ^{15}N labelled compound **2** and the corresponding PDI(Me)$_8$-2P COF. Compound **2** shows strong signals at -47 and -50 ppm (the splitting could be due to molecular packing in the solid state). When this compound is converted to the COF, however, the ^{15}N signal disappears, confirming complete exchange of the aniline modulator for the interconnecting benzidine.

L. Thermogravimetric analysis

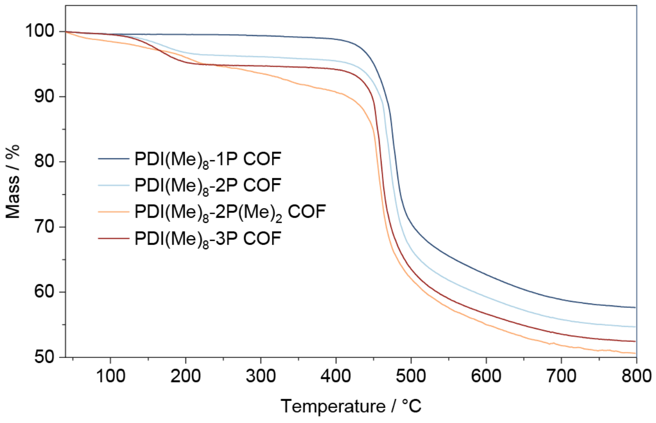

Figure S10. Thermogravimetric (TG) analysis of the PDI COFs, recorded at 5 K min^{-1} in argon atmosphere. Desorption of guest molecules is observed for the larger-pore PDI(Me)$_8$-2P, -2P(Me)$_2$, and -3P COFs between 100 and 200 °C. All COFs are thermally stable up to above 400 °C. A sharp mass loss step is observed at 460 °C for the more flexible -2P(Me)$_2$ and 3P COFs. This is shifted to 470 °C and 480 °C, respectively, for the more rigid -2P and -1P COFs.

M. N_2 sorption

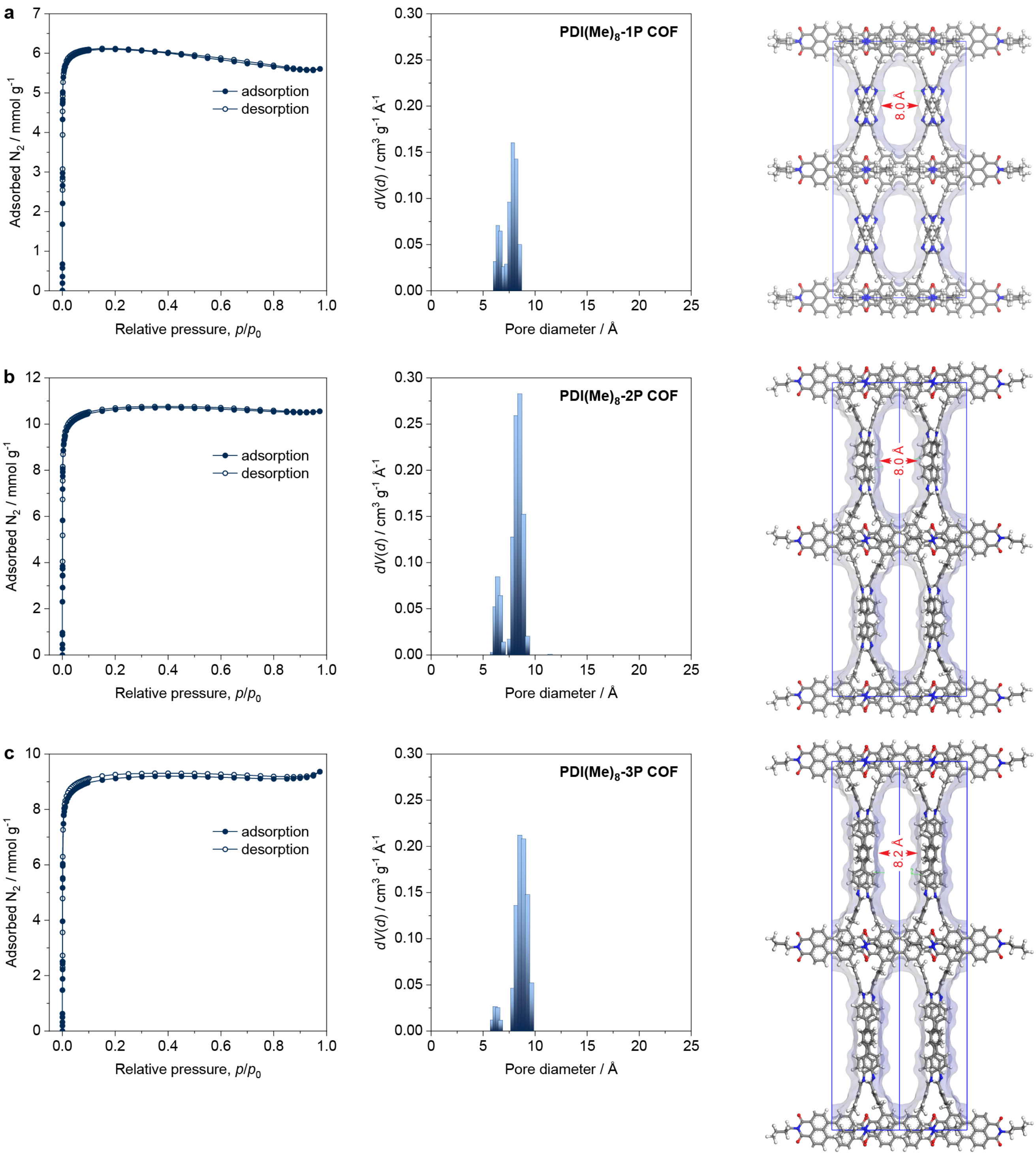

Figure S11. Nitrogen sorption isotherms recorded at 77 K of the (**a**) PDI(Me)$_8$-1P COF, (**b**) PDI(Me)$_8$-2P COF, and (**c**) PDI(Me)$_8$-3P COF. Left panel: The COFs display type I isotherms that are characteristic of microporous materials. Middle: Pore size distributions (PSDs) were obtained by fitting the desorption branch of the isotherms using the quenched solid density functional theory (QSDFT) model for carbon with slit/cylindrical pores. The COFs show very narrow bimodal PSDs with main pore sizes of 8.0 Å, 8.4 Å and 8.7 Å for the -1P, -2P and -3P COFs, respectively, that correspond very well to the wall-to-wall distances of the slit-like pores in the COFs (right panel). The second peak in the PSDs around 6 Å is attributed to the “pockets” at the corners of the slit pores.

N. Optical spectroscopy – PDI monomer

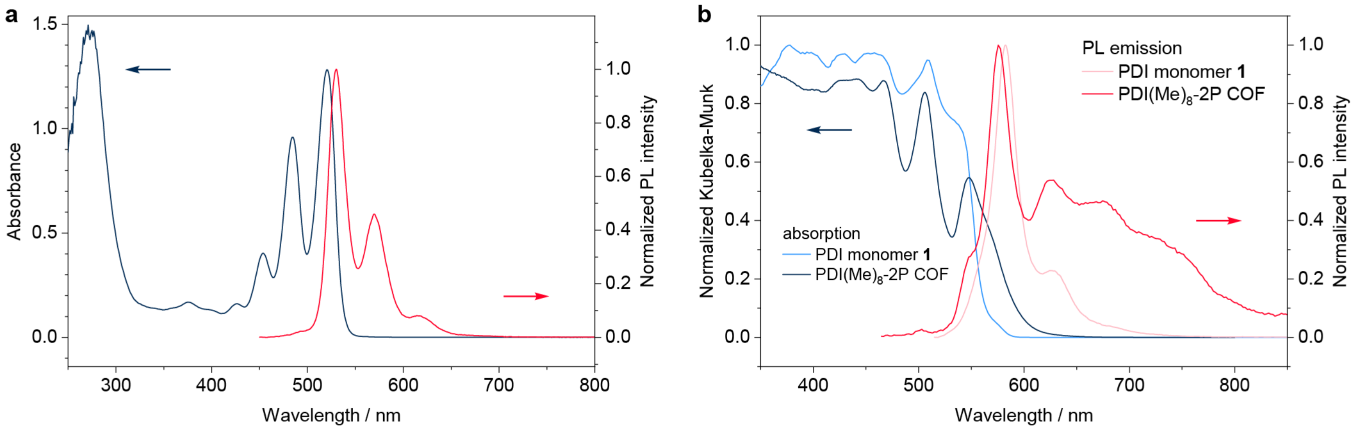

Figure S12. (**a**) UV-Vis absorption spectrum of a 10 µM solution of the PDI building block (compound **1**) in chloroform (black) and corresponding PL emission spectrum (0.1 µM solution, λ_{exc} = 420 nm). Due to its rigidity, the compound has a very small Stokes shift of 10 nm (530 nm vs. 520 nm). (**b**) Comparison of the absorption and emission spectra of the PDI monomer **1** in the solid state and the PDI(Me)$_8$-2P COF. Compound **1** has a slightly red-shifted emission and lacks the broad excimer-like emission around 700 nm due to different packing of the PDI moieties.

O. PL mapping of a PDI(Me)$_8$-2P COF crystal

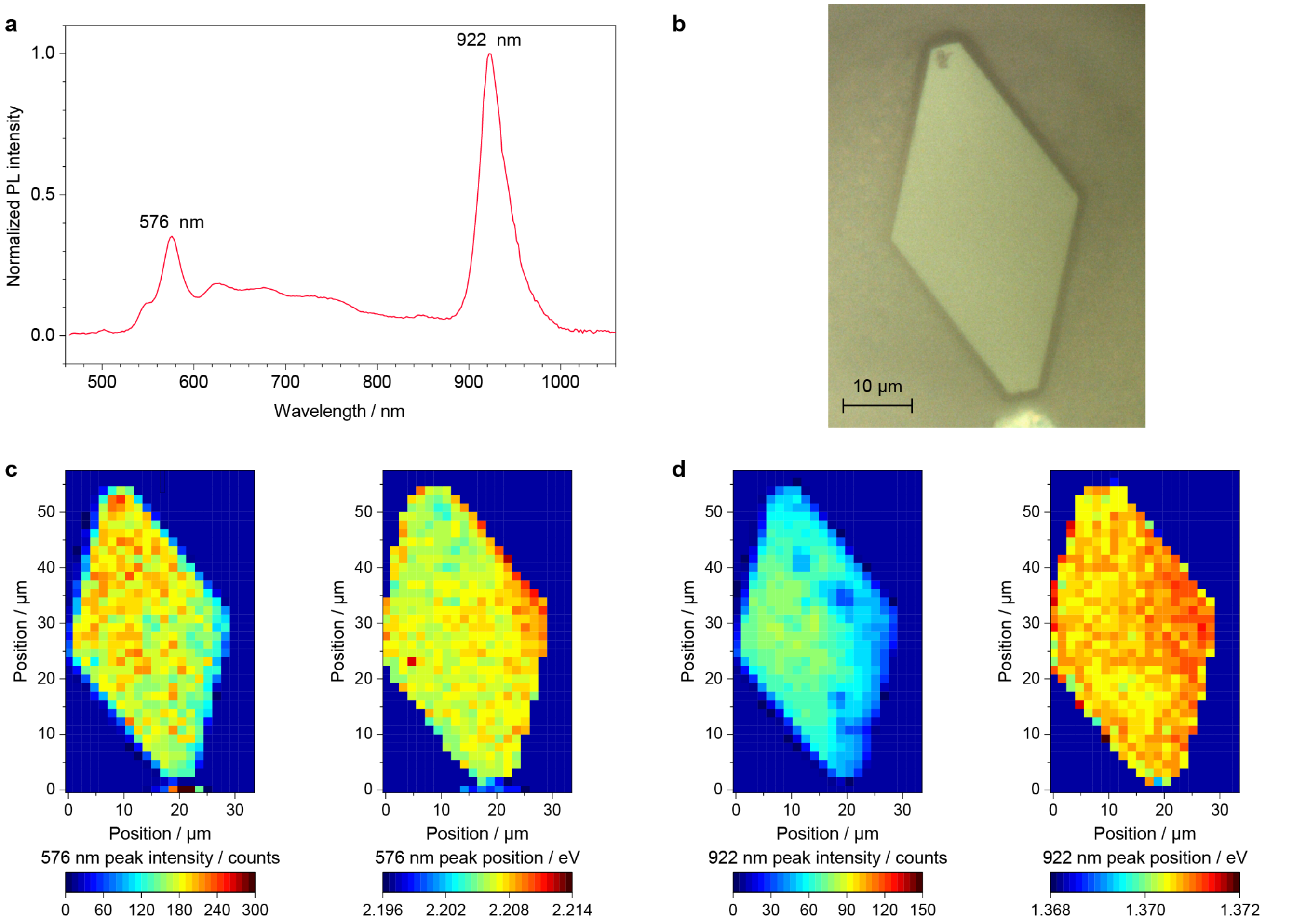

Figure S13. Homogeneity of the PL properties. (**a**) PL emission spectrum of the PDI(Me)$_8$-2P COF. (**b**) Optical microscope image of the COF crystal used for the spectroscopic studies. (**c**) Maps showing the distribution of emission intensity (left panel) and the spectral position of the peak (right panel) of the 576 nm PL across the COF crystal. (**d**) The same maps for the 922 nm PL. The optical properties are very homogeneous across the crystal. The peak intensities are slightly reduced on the lower right side of the crystal due to weak defocussing.

P. PL spectroscopy – PDI(Me)$_8$-1P, PDI(Me)$_8$-2P(Me)$_2$, and PDI(Me)$_8$-3P COFs

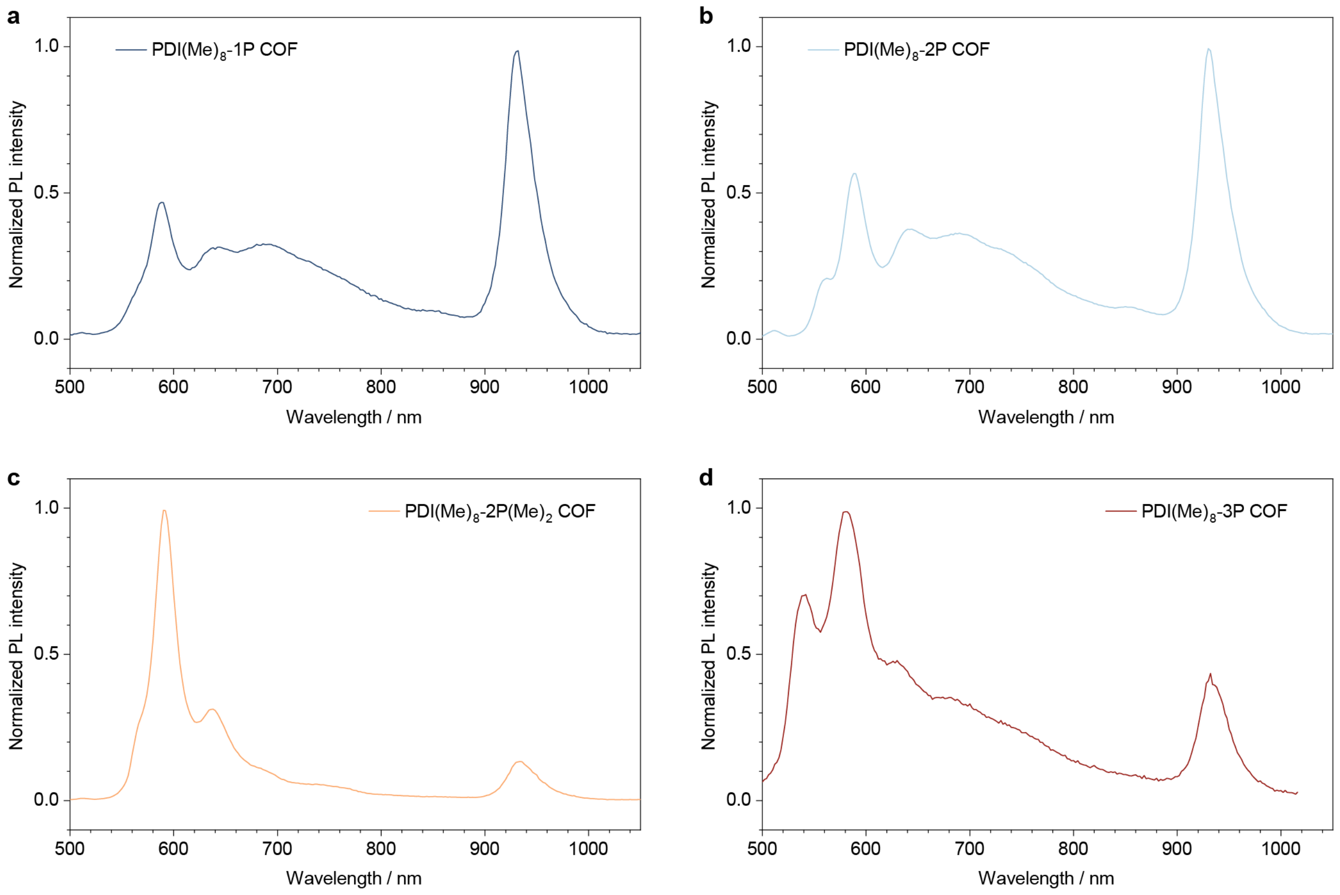

Figure S14. Steady-state PL spectra of the PDI COFs. (**a**) PDI(Me)$_8$-1P COF, (**b**) PDI(Me)$_8$-2P COF, (**c**) PDI(Me)$_8$-2P(Me)$_2$ COF, (**d**) PDI(Me)$_8$-3P COF. The smaller and more rigid -1P and -2P COFs show more pronounced broad excimer-like emission in the 650 – 750 nm range, and triplet emission at 920 nm. In contrast, the -2P(Me)$_2$ and -3P COFs display more monomer-like PL with sharp singlet emission at 580 nm and only weak phosphorescence.

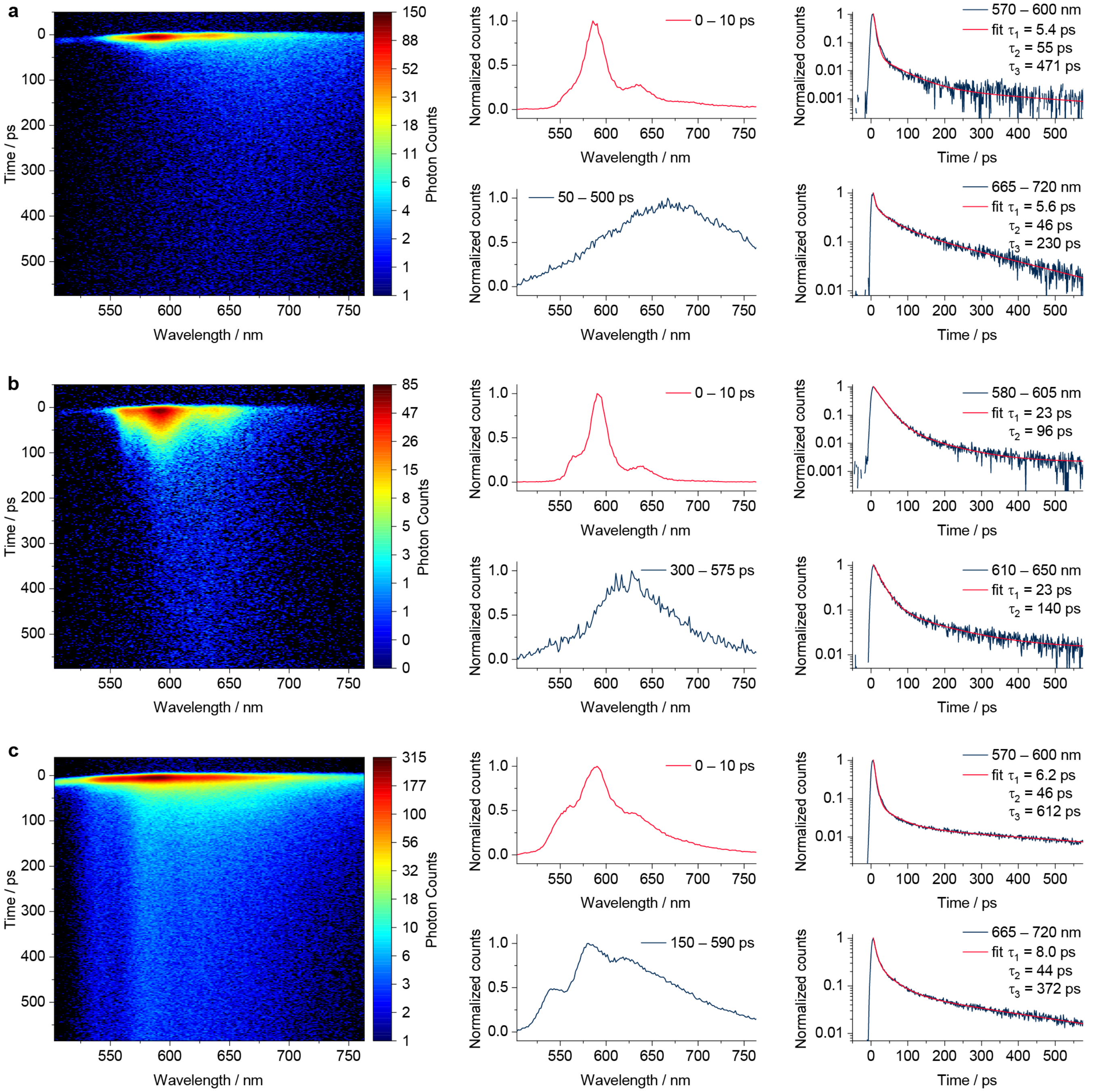

Figure S15. Time-resolved PL of the PDI COFs, captured with a streak camera. (**a**) PDI(Me)$_8$-1P COF, (**b**) PDI(Me)$_8$-2P(Me)$_2$ COF, (**c**) PDI(Me)$_8$-3P COF. The middle panels show the decay-associated spectra integrated from 0 – 10 ps (top) and later times (bottom) for each COF. The right panels show the PL decay transients integrated over the position of the sharp singlet emission (top) and the broad excimer-like emission (bottom).

Q. TAPB-DMPDA COF

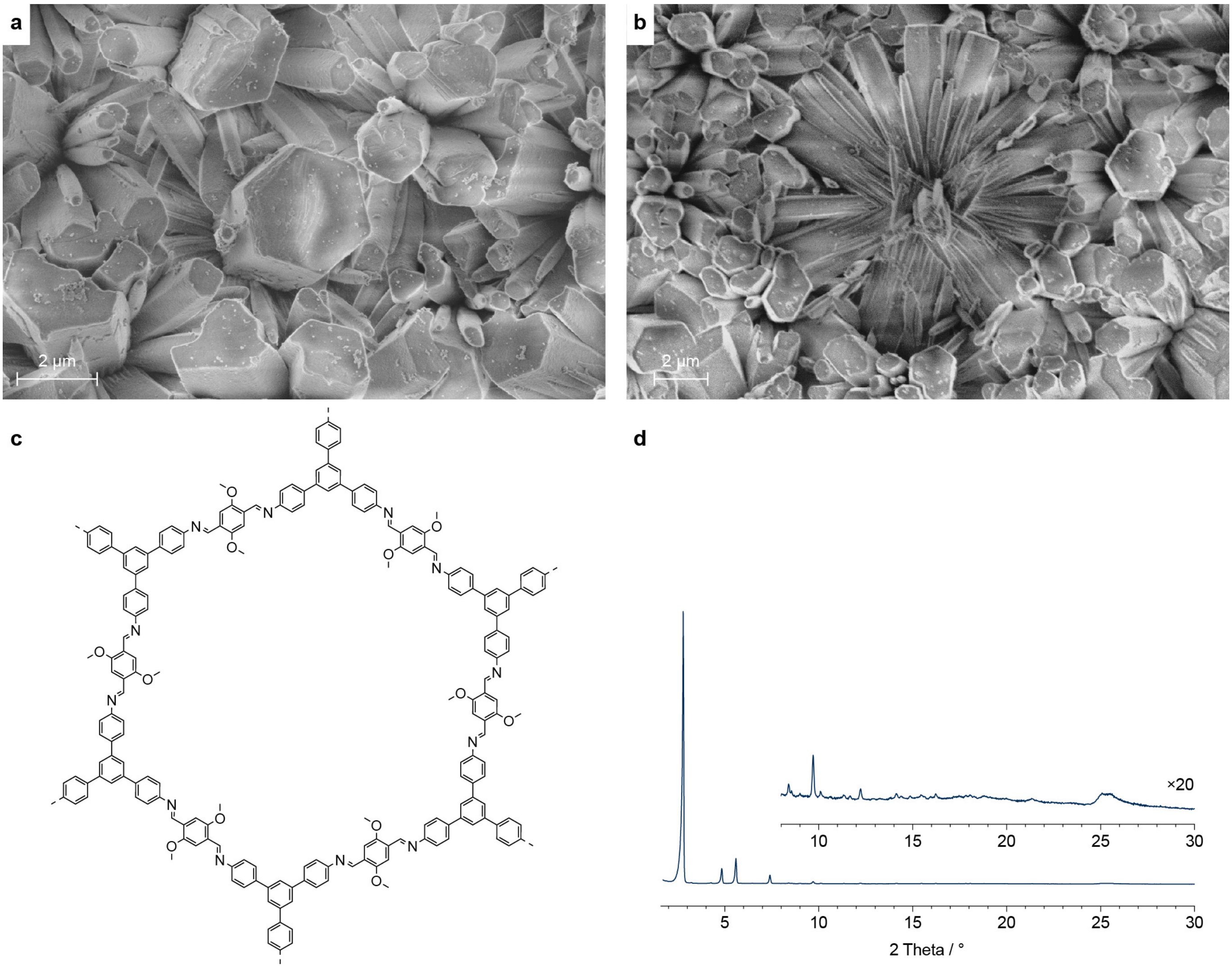

Figure S16. Synthesis of large-crystal TAPB-DMPDA COF via our high-temperature double-modulator protocol. (**a**,**b**) This COF crystallizes as hexagonal needles with up to 3.3 µm width and 6 µm length. (**c**) Chemical structure of the COF. (**d**) PXRD pattern, highlighting the very high crystallinity of the material.

R. References

[1] ACD/C+H NMR Predictors and DB 2025.1.2, Advanced Chemistry Development, Inc., Toronto, ON, Canada, www.acdlabs.com, 2025.

[2] T. N. Blanton, M. Rajeswaran, P. W. Stephens, D. R. Whitcomb, S. T. Misture, J. A. Kaduk, Crystal structure determination of the silver carboxylate dimer [Ag(O2C22H43)]2, silver behenate, using powder X-ray diffraction methods, *Powder Diffraction* **2012**, *26*, 313.

[3] L. W. Finger, D. E. Cox, A. P. Jephcoat, A Correction for Powder Diffraction Peak Asymmetry due to Axial Divergence, *J. Appl. Chrystallogr.* **1994**, *27*, 892.

[4] T. Düren, F. Millange, G. Férey, K. S. Walton, R. Q. Snurr, Calculating Geometric Surface Areas as a Characterization Tool for Metal-Organic Frameworks, *J. Phys. Chem. C* **2007**, *111*, 15350.

[5] T. Teraoka, S. Hiroto, H. Shinokubo, Iridium-Catalyzed Direct Tetraborylation of Perylene Bisimides, *Org. Lett.* **2011**, *13*, 2532. https://doi.org/10.1021/ol2004534

[6] A. Natraj, W. Ji, J. Xin, I. Castano, D. W. Burke, A. M. Evans, M. J. Strauss, M. Ateia, L. S. Hamachi, N. C. Gianneschi, Z. A. ALOthman, J. Sun, K. Yusuf, W. R. Dichtel, Single-Crystalline Imine-Linked Two-Dimensional Covalent Organic Frameworks Separate Benzene and Cyclohexane Efficiently, *J. Am. Chem. Soc.* **2022**, *144*, 19813. https://doi.org/10.1021/jacs.2c07166
